# Inhibition of low-density lipoprotein receptor degradation with a cyclic peptide that disrupts the homodimerization of IDOL E3 ubiquitin ligase†
†Electronic supplementary information (ESI) available. See DOI: 10.1039/c8sc01186a


**DOI:** 10.1039/c8sc01186a

**Published:** 2018-06-26

**Authors:** Eilidh K. Leitch, Nagarajan Elumalai, Maria Fridén-Saxin, Göran Dahl, Paul Wan, Paul Clarkson, Eric Valeur, Garry Pairaudeau, Helen Boyd, Ali Tavassoli

**Affiliations:** a Chemistry , University of Southampton , Southampton , SO17 1RE , UK . Email: a.tavassoli@soton.ac.uk; b Medicinal Chemistry , Cardiovascular and Metabolic Diseases , IMED Biotech Unit , AstraZeneca , Pepparedsleden 1 , Mölndal , 43150 , Sweden; c Structure and Biophysics , Discovery Sciences , IMED Biotech Unit , AstraZeneca , Pepparedsleden 1 , Mölndal , 43150 , Sweden; d AstraZeneca , Cambridge Science Park, 310 Milton Rd , Cambridge , CB4 0FZ , UK; e Drug Safety and Metabolism , IMED Biotech Unit , AstraZeneca , Pepparedsleden 1 , Mölndal , 43150 , Sweden; f Institute for Life Sciences , University of Southampton , Southampton , SO17 1BJ , UK

## Abstract

Cellular uptake of circulating cholesterol occurs *via* the low density lipoprotein receptor (LDLR). The E3 ubiquitin ligase IDOL is a mediator of LDLR degradation, with IDOL homodimerization thought to be required for its activity. To probe the possibility of modulating LDLR levels with an inhibitor of IDOL homodimerization, we screened a SICLOPPS library of 3.2 million cyclic peptides for compounds that disrupt this protein–protein interaction. We identified *cyclo*-CFFLYT as the lead inhibitor, and improved its activity through the incorporation of non-natural amino acids. The activity of the optimized cyclic peptide was assessed in hepatic cells, with a dose-dependent increase in LDLR levels observed in the presence of our IDOL homodimerization inhibitor.

## Abstract 2

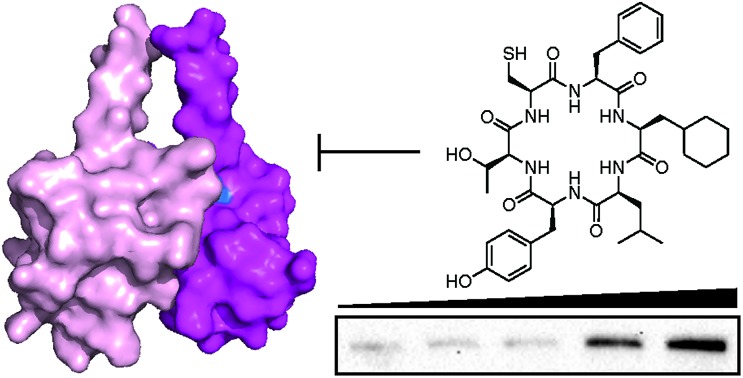
A cyclic peptide IDOL homodimerization inhibitor identified from a genetically encoded SICLOPPS library is active *in vitro* and in cells.

## Introduction

Cholesterol is an essential component of the plasma membrane, providing fluidity, forming lipid rafts, functioning to concentrate signalling molecules, and as a key pre-cursor for steroid hormone synthesis. Cells obtain cholesterol *via de novo* synthesis (a complex and multi-step pathway), or by uptake from circulating lipoproteins; both these routes are tightly regulated at the transcriptional and post-transcriptional levels. Despite its critical cellular functions, excess cholesterol levels within the plasma are deleterious to human health, leading to the formation of atherosclerotic plaques and subsequent development of cardiovascular disease.^1^ Cellular uptake of circulating cholesterol primarily occurs *via* the low density lipoprotein receptor (LDLR).^2^ LDLR is expressed in liver cell membranes and removes circulating cholesterol-carrying LDLs from the plasma *via* receptor mediated endocytosis.^3^,^4^ Reduced LDLR activity is a contributing factor to the development of hypercholesterolemia across the general population, and is thought to cause just over half of all ischemic heart disease cases worldwide.^5^ Transcriptional regulation of LDLR occurs *via* sterol regulatory element binding proteins (SREBPs),^6^ which are expressed as precursors that are activated upon cleavage by a series of proteases in response to decreased cellular sterol levels. Activated SREBPs promote the expression of several target genes involved in cholesterol biosynthesis and uptake, including LDLR.^7^ Conversely, the liver X receptors (LXRs) co-ordinate the transcriptional response to elevated cellular cholesterol levels. The activation of LXRs by oxysterol ligands^8^ increases transcription of genes whose protein products work to reduce intracellular cholesterol levels.^9^,^10^ These include proteins responsible for cellular efflux, transport and excretion of cholesterol, as well as a ubiquitin ligase named the inducible degrader of LDLR (IDOL), that triggers degradation of LDLR *via* the lysosomal pathway.^11^

IDOL is a unique RING-type E3 ubiquitin ligase, containing both an E3 RING and a FERM domain.^12^ The E3 ligase activity of IDOL promotes poly-K63 and poly-K48-ubiquitination^13^ of the cytoplasmic tail of LDLR, while its FERM domain binds directly to the cytoplasmic tail of LDLR, providing specific targeting, as well as providing hydrostatic interactions that anchor IDOL at the intracellular surface of the plasma membrane.^14^ IDOL also autocatalyzes its own ubiquitination and degradation. Functional IDOL is a homodimer that is formed *via* a protein–protein interaction (PPI) between its RING domain, with a buried surface area of 1862 Å^2^.^15^ Structure-guided mutational studies have shown that a V431R/L433R dimer defective mutant is unable to facilitate IDOL induced LDLR degradation, as well as autocatalyzed IDOL degradation.^15^ Furthermore, IDOL null cells have been shown to be unresponsive to LXR agonists; despite having lower *LDLR* mRNA levels, these cells display a higher basal level of LDLR protein than wild type cells, which leads to elevated uptake of LDL.^16^

Two posttranslational regulators of LDLR have been identified as potential targets for therapeutic intervention. The first is PCSK9, which binds to the EGF-A repeat of LDLR and leads to the lysosomal degradation of LDLR. This processes is targeted for therapeutic intervention by the monoclonal antibodies ecolocumab and alicrocumab, both approved for the treatment of hypercholesterolemia.^17^,^18^ The second is IDOL, a target gene of LXRs, which are activated by oxysterol ligands under high cellular sterol conditions. Since its discovery in 2009,^11^ mounting genetic evidence suggests that IDOL is a viable pharmacological target for the treatment of hypercholesterolemia.^19^ However, no compounds have been reported to date that are capable of inhibiting IDOL mediated LDLR degradation. Such a molecule would not only serve as a chemical tool to validate the therapeutic potential of IDOL inhibition, it could also serve as the starting point for the development of a therapeutic agent. Given our expertise in identifying and developing cyclic peptide inhibitors of PPIs,^20^–^22^ we sought to identify an inhibitor of the homodimeric PPI of the IDOL RING domain.

## Results

### Identification of cyclic peptide IDOL homodimerization inhibitors

**Fig. 1:**
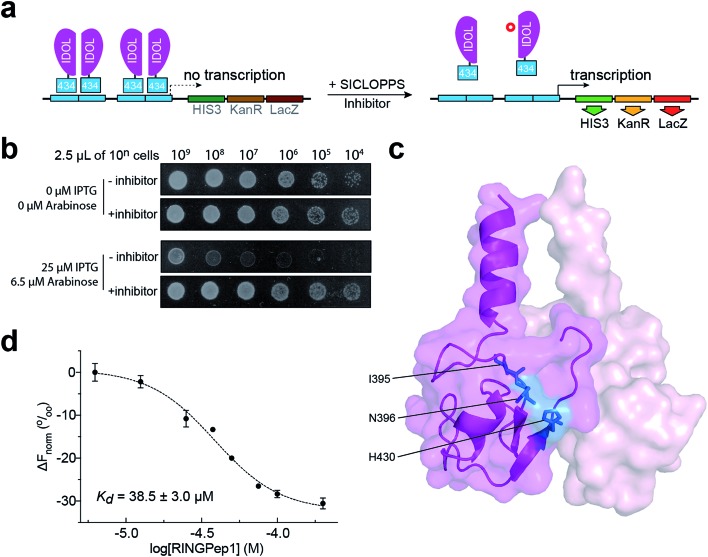
Identification of RINGPep1 from a SICLOPPS library. (a) Schematic of IDOL RTHS. The expression of the 434-IDOL fusion protein is induced by IPTG. IDOL dimerization leads to the formation of a functional repressor that prevents expression of the 3 reporter genes downstream: *His3* (imidazole glycerol phosphate dehydratase), *KanR* (aminoglycoside 3′-phosphotransferase, kanamycin resistance), and *LacZ* (β-galactosidase). A cyclic peptide inhibitor of IDOL homodimerization will disrupt the repressor, leading to expression of the reporter genes and survival of the host colony on selective media. (b) Drop spotting analysis of RINGPep1 activity in the IDOL RTHS; serial dilutions (2.5 μL of ∼10^*n*^ cells per mL) of the IDOL RTHS and the IDOL RTHS containing a SICLOPPS plasmid encoding RINGPep1. In the absence of IPTG and arabinose the strains have full growth capability, however upon addition of 25 μM IPTG (inducer of 434-IDOL) and 6.5 μM arabinose (inducer of SICLOPPS) the IDOL RTHS displays inhibition of growth, which is restored in the strain containing the plasmid encoding RINGPep1. (c) The residues on IDOL perturbed by RINGPep1 binding identified by ^1^H ^15^N HSQC NMR are shown in blue (for spectrum see ESI Fig. 5,† for additional views of the structure, see ESI Fig. 6†). Each monomer of the IDOL homodimer is shown in a different shade of pink. (d) Assessing the binding of RINGPep1 to IDOL by MST reveals a *K*_d_ of 38.5 ± 3.0 μM. Data represented as mean ± SEM, *n* = 3.

We used a previously reported genetically encoded high-throughput screening platform that combines a bacterial reverse two-hybrid system (RTHS)^21^,^23^–^25^ with a plasmid-encoded library of 3.2 million cyclic hexapeptides using split intein circular ligation of peptides and proteins (SICLOPPS).^26^,^27^ We began by constructing a bacterial RTHS for IDOL homodimerization. IDOL is expressed as an N-terminal fusion with the 434 bacteriophage repressor, with the 38 amino acid disordered region of the 434 repressor acting as linker. IDOL homodimerization is expected to bring together two 434 proteins to form a functional repressor that binds to the operator sites engineered onto the *E. coli* chromosome, and prevent the expression of 3 genes downstream that are required for growth and survival on selective media (Fig. 1a). We verified the formation of a functional repressor and suppression of growth on selective media upon IDOL homodimerization by drop-spotting (Fig. 1b), and by ONPG assay (ESI Fig. 1†) as previously detailed.^21^ A SICLOPPS CXXXXX library (where X = any canonical amino acid) was prepared as previously detailed^28^ and transformed into the IDOL RTHS prior to plating on selective media. After incubation of the selection plates for 48 h, 480 surviving colonies were picked and individually assessed for arabinose-dependent survival on selective media by drop-spotting. The 64 colonies showing the expected phenotype were subjected to secondary screening by isolating the SICLOPPS plasmids contained within, and assessing their ability to restore survival in an otherwise identical RTHS for an unrelated protein homodimer (C-terminal binding protein)^20^ to eliminate any false positives and non-specific binders. The 43 SICLOPPS plasmids that did not affect survival of the unrelated RTHS were ranked by drop-spotting. This ranking process resulted in 6 peptides that proved significantly more active than others in the cohort (Fig. 1b). These SICLOPPS plasmids were sequenced to reveal the identity of the potential cyclic peptide IDOL homodimerization inhibitors (Table 1). We observed some homology in the sequence of these 6 cyclic peptides; 5 contained a T or S next to the set cysteine residue, while 5 of the selected hits contained an aliphatic amino acid (V, L or I) as residue 3 in the cyclic peptide. In all peptides containing a T, there is an F two residues away. Further homology was also visible in the hits; 5 cyclic peptides contained a tripeptide comprised of an aromatic amino acid (F or Y) followed by two aliphatic amino acids (L or I). Interestingly, *cyclo*-CTIFLL and *cyclo*-CTLILF contain the same amino acid residues, appearing in a different order. The 6 lead peptides were synthesized by SPPS in order to carry out *in vitro* binding analysis.

**Table 1:** Affinities obtained by SPR for the cyclic peptides hits identified in our screen^*a*^

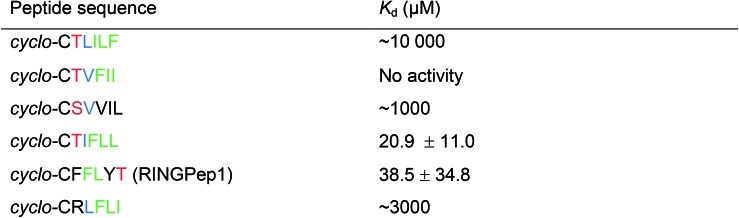

^*a*^Colour coding: T or S residues are shown in red, I/L–I/L–F/Y motifs shown in green, aliphatic residues in position 3 shown in blue.

### Assessing the activity of the identified inhibitors *in vitro*

We initially assessed the affinity of the 6 selected cyclic peptides for IDOL by surface plasmon resonance (SPR). Although saturation at the highest concentrations was not observed, due to hydrophobic effects, *K*_d_ values could be determined for five peptides (Table 1). The most potent cyclic peptides *cyclo*-CTIFLL and *cyclo*-CFFLYT demonstrated *K*_d_ values of 20.9 ± 11.0 μM and 38.5 ± 34.8 μM respectively (ESI Fig. 2†). The hit rate for our IDOL screen is in line with that for previous SICLOPPS screens.^21^,^25^ It should be noted that although *cyclo*-CTIFLL and *cyclo*-CTLILF contain the same amino acids, changing the order in which these are arranged results in activity loss. The linear counterparts of CTIFLL and CFFLYT were also assessed for binding to IDOL by SPR, with no binding observed for either linear peptide (ESI Fig. 3†), suggesting that a cyclic backbone is required for activity.

The lead candidates, *cyclo*-CTIFLL and *cyclo*-CFFLYT, were next analyzed for binding to the RING domain of IDOL by ^1^H ^15^N HSQC NMR. We also assessed the binding of each peptide to the FERM domain of IDOL as a negative control. We did not observe any chemical shift changes within either domain with *cyclo*-CTIFLL (ESI Fig. 4†). However, incubation with *cyclo*-CFFLYT with the RING domain of IDOL resulted in modest chemical shift changes for 3 amino acids (Ile395, Asn396 and His430), indicating the cyclic peptide interacts with this protein without disrupting its overall structure. No changes were observed in the chemical shifts upon incubation of either cyclic peptide with the FERM domain of IDOL (ESI Fig. 5†). It should be noted that the 3 residues affected by *cyclo*-CFFLYT (named RINGPep1) form part of the PPI interface in the crystal structure of the homodimerized RING domains of IDOL (Fig. 1c and ESI Fig. 6†).^15^ Furthermore, mutation of V431R coupled with a L433R mutation is known to result in a dimer deficient IDOL.^15^

We further assessed the binding affinity of RINGPep1 for the RING domain of IDOL by Microscale Thermophoresis (MST), measuring a *K*_d_ of 38.5 ± 3.0 μM (Fig. 1d), in line with the value measured by SPR.

### Improving the activity of RINGPep1

**Fig. 2:**
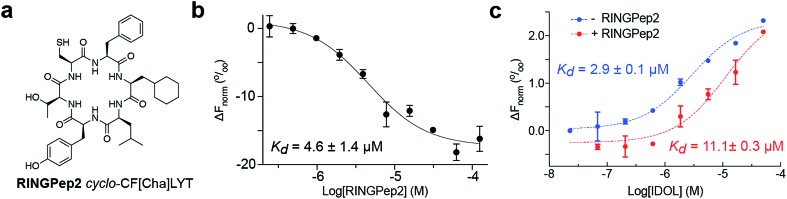
Characterizing the activity of RINGpep2. (a) Structure of RINGPep2 (*cyclo*-CF-Cha-LYT). (b) Binding of RINGpep2 to IDOL as measured by MST (*K*_d_ = 4.6 ± 1.4 μM). (c) RINGPep2 (100 μM) increases the apparent *K*_d_ of IDOL homodimerization from 2.9 ± 0.1 μM to 11.1 ± 0.3 μM. Data represented as mean ± SEM, *n* = 3.

**Fig. 3:**
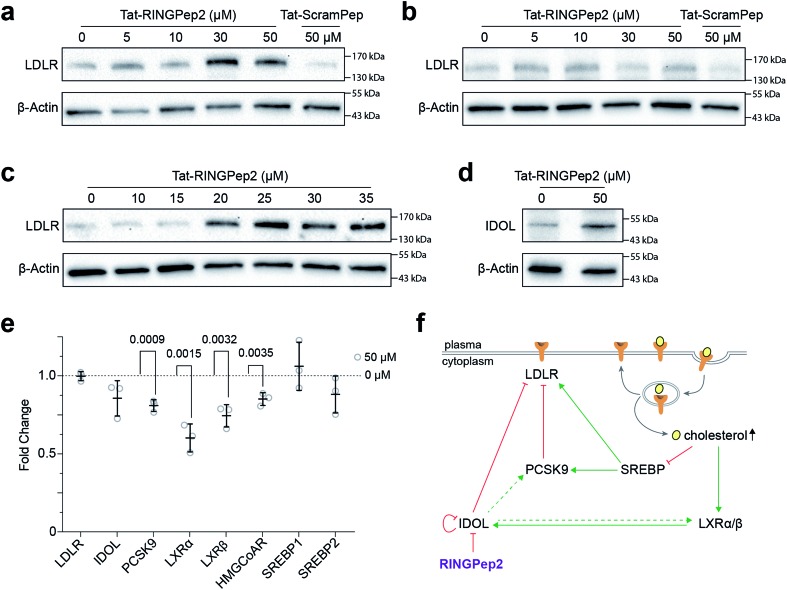
Assessing Tat-RINGPep2 activity in hepatocyte cells. (a) HepG2 cells were treated with Tat-RINGPep2 (0–50 μM) or Tat-ScramPep (50 μM) for 48 h in serum rich media followed by 24 h in serum free media. (b) ΔIDOL HepG2 cells were treated with tat-RINGPep2 (0–50 μM) or Tat-ScramPep (50 μM) as above. (c) HepG2 cells were treated with Tat-RINGPep2 (0–35 μM) as above to determine a cellular EC_50_. (d) HepG2 cells were treated with 0 μM or 50 μM Tat-RINGPep2 as above. (e) HepG2 cells were treated with 0 μM or 50 μM Tat-RINGPep2 for 72 h in lipoprotein depleted serum and mRNA levels detected by RT-qPCR. Genes expression levels were normalised to 18S and β-actin levels. Data is shown as fold change of Tat-RINGPep2-treated samples *versus* untreated cells. All data shown as mean ± SEM, *n* = 3, *p* values for significant changes (*t*-test) are shown on the graph. (f) Graphical representation of the LDLR regulatory pathways, and an overview of the effect of RINGPep2 observed in cells.

We sought to improve the affinity of RINGPep1 prior to assessing its effect in cells. We used alanine-scanning to identify residues that were critical to the activity of RINGpep1; we constructed 5 SICLOPPS plasmids where each amino acid (except the set cysteine, which is required for intein splicing) was replaced with alanine. These plasmids were transformed into the IDOL RTHS and assessed for disruption of IDOL homodimerization by drop-spotting. Only cells containing *cyclo*-CFALYT did not grow on selective media (ESI Fig. 7†) suggesting that the second phenylalanine residue is essential for the activity of the peptide. We therefore synthesized a handful of RINGPep1 analogues with the second position containing a non-natural phenylalanine analogue. After purification, the affinity of each of these compounds for IDOL was assessed by MST. The most potent analogues in this series were those containing 3-cyclohexyl-l-alanine (Cha) or 3-(1-naphthyl)-l-alanine (ESI Fig. 8†). Given its marginally better water solubility, we chose to take forward the Cha derivative (named RINGpep2, Fig. 3a). RINGPep2 bound to full length IDOL by MST with an affinity of 4.6 ± 1.4 μM (Fig. 2b). The ability of RINGpep2 to disrupt IDOL homodimerization was assessed *in vitro* using a competition assay in which increasing concentrations of IDOL were titrated into a 45 nM solution of dye-labelled IDOL. Binding of non-labelled IDOL to dye-labelled IDOL will be observed in the MST signal, allowing the *K*_d_ of IDOL homodimerization to be quantified. Using this approach, we measured a *K*_d_ of 2.9 ± 0.1 μM for IDOL homodimerization. The experiment was repeated in the presence of RINGpep2 (100 μM), with the expectation that the apparent *K*_d_ of IDOL homodimerization would increase in the presence of an inhibitor of IDOL homodimerization. In line with this, we measured an apparent *K*_d_ for IDOL homodimerization of 11.1 ± 0.3 μM in the presence of RINGPep2 (Fig. 2c), indicating that this compound disrupts the homodimerization of IDOL. As a control, a scrambled derivative (*cyclo*-CYLFT[Cha] ScramPep) was synthesized, which did not bind to IDOL by MST (ESI Fig. 9†).

The effect of RINGPep2 on the homodimerization-dependent autoubiquitination of IDOL was assessed *in vitro*. IDOL was incubated with E1, UBE2D2, ubiquitin and ATP and autoubiquitnation of IDOL (a feature of ring-type E3 ligases) was observed by western blot analysis (ESI Fig. 10†). RINGPep2 showed poor solubility in the assay buffer, thus preincubation of IDOL with up to 100 μM of RINGPep2 did not affect IDOL ubiquitination in this assay (ESI Fig. 10†). However, when the experiment was repeated with a Tat-conjugated RINGPep2 (the Tat-tag aids solubility), we observed inhibition of IDOL autoubiquitination with 10 μM of Tat-tagged RINGpep2 (ESI Fig. 10†). Taken together, the above data demonstrates that RINGPep2 binds to IDOL and disrupts its homodimerization.

### Assessing the activity of RINGPep2

We used RINGPep2 to probe the effect of inhibiting IDOL homodimerization in HepG2 cells. While this compound is not drug-like in its current form, it may still be used as tool to probe the role of IDOL in cells, and to assess the potential of targeting LDLR degradation *via* perturbation of IDOL homodimerization. In order to assist their permeability into cells, both RINGPep2 and ScramPep were conjugated to a Tat-tag, attached *via* a disulfide bond between the invariable Cys of our cyclic peptides and a Cys residue introduced position 1 of the Tat peptide (to give Tat-RINGPep2 and Tat-ScramPep).^25^ It should be noted that this disulphide bond is believed to be reduced during internalization leading to release of the untagged cyclic peptide into the cytoplasm.

We observed a dose dependent increase in LDLR protein in HepG2 cells treated with increasing concentrations of Tat-RINGPep2, with no effect from Tat-ScramPep (Fig. 3a), indicating that the response is both specific to the sequence of RINGPep2 and independent of any effect of the Tat-tag. To probe the IDOL-specificity of the observed effect, we used a knockout HepG2 cell line, lacking functional IDOL using CRISPR/Cas9 technology (ΔIDOL HepG2).^29^ Interestingly, we observed no effect from RINGPep2 on LDLR levels in the ΔIDOL HepG2 cells (Fig. 3b), demonstrating that the observed activity of RINGPep2 is dependent on to the presence of functional IDOL in cells. We also used RINGPep2 to assess the role of IDOL homodimerization on IDOL autodegradation. The EC_50_ of Tat-RINGPep2 in HepG2 cells was measured *via* its effect on LDLR levels as between 15 to 20 μM (Fig. 3c). In line with previous reports of IDOL dimerization inhibition,^15^ we observed increased IDOL levels in cells treated with Tat-RINGPep2 (Fig. 3d). We also assessed the toxicity of Tat-RINGPep2 and Tat-ScramPep to HepG2 cells and ΔIDOL HepG2 cells using a commercial luminescence-based toxicity assay; we observed no toxicity from compound in either cell line at concentrations up to 100 μM (ESI Fig. 11†).

The effect of Tat-RINGPep2 on the transcription of several genes involved in the regulatory pathways of LDLR (*LDLR*, *IDOL*, *PCSK9*, *LXRα*, *LXRβ*, *HMGCoAR*, *SREBP1* and *SREBP2*) was investigated by qPCR. We observed no effect on the transcription of *LDLR*, *IDOL*, *SREBP1*, or *SREBP2* from Tat-RINGPep2 (50 μM), whereas a decrease in mRNA levels of the *PCSK9*, *LXRα*, *LXRβ*, and *HMGCoAR* were observed (Fig. 3e). While the effect of increased LDLR levels on the transcription of *PCSK9* and *HMGCoAR* are in line with previous reports,^30^ we also observed a previously unreported effect on *LXRα* and *LXRβ* expression. As IDOL is a target gene of the LXR transcription factors, it is likely that the observed down regulation of these genes is as a consequence of a previously unidentified auto-regulatory circuit for IDOL. The link between IDOL and *LXR* transcription was further verified by comparing *LXRα* levels in HEPG2 cells with that in the ΔIDOL HepG2 cells. We observed ∼50% less *LXRα* mRNA in the ΔIDOL HepG2 cells (ESI Fig. 12a†), in line with the reduction of *LXRα* mRNA observed in HepG2 cells treated with Tat-RINGPep2 (Fig. 3e). As a further control, we assessed the effect of Tat-RINGPep2 (50 μM) on the transcription of *LXRα* in ΔIDOL HepG2 cells. We did not observe a significant difference in *LXRα* transcription between ΔIDOL HepG2 cells treated with Tat-RINGPepe2 and untreated cells (ESI Fig. 12b†).

## Discussion

There continues to be significant potential in targeting protein–protein interactions for therapeutic purpose. Identifying inhibitors against this target class remains highly challenging, however, there has been significant progress in recent years.^31^–^33^ A key challenge for the field remains the production of chemical entities that may be used to validate genetic data on the therapeutic potential of a given PPI.^34^ Such compounds, if generated and validated relatively rapidly, would inform key decisions on whether to initiate a drug discovery campaign against a given PPI. Cyclic peptides have proven to be a particularly good scaffold for this purpose, with several available methodologies for their rapid generation and screening.^22^,^35^ And while the translation of cyclic peptides to the clinic has proven challenging, advances continue to be made in converting these compounds to more drug-like molecules.^36^

To illustrate the above, we used a genetically encoded high-throughput screening platform to screen a library of 3.2 million cyclic peptides for inhibitors of IDOL homodimerization. Despite the ubiquitin proteasome system, and E3 ubiquitin ligases in particular, being very challenging targets,^37^ 6 cyclic peptides were identified from our screen. The activity of the lead peptide (*cyclo*-CFFLYT, RINGPep1) was verified *in vitro*; in order to improve the potency of the original hit, we synthesized and tested derivatives containing non-natural phenylalanine derivatives in position 3 of the ring (identified as being critical for activity through alanine scanning). This resulted in a 7-fold improvement in IDOL binding and a compound (RINGPep2) that was sufficiently potent for assessment in cells. We demonstrated that RINGPep2 inhibits both IDOL mediated LDLR degradation and IDOL autodegradation in hepatocytes, with an EC_50_ of ∼20 μM. This effect was shown to be IDOL specific, with no activity observed from RINGPep2 in a cell line with deleted IDOL functionality. This finding substantiates previous reports that the generation of a dimer defective IDOL was unable to elicit IDOL mediated LDLR degradation.^15^ Further, our data demonstrates the viability of targeting this pathway with a compound that inhibits IDOL homodimerization.

We also investigated the impact of the increase in LDLR protein concentration caused by IDOL homodimerization inhibition on the expression of a handful of genes involved in the LDLR regulatory pathway. We observed downregulation of *PCSK9* and *HMGCoAR* in response to RINGPep2; both these genes are targets of SREBP proteins whose activity is inhibited by the increase in cellular sterol uptake arising from increased LDLR levels (Fig. 3f). *HMGCoAR* encodes the rate limiting enzyme in *de novo* cholesterol biosynthesis, while PCSK9 is a post-translational regulator of LDLR. Regulation of LDLR by PCSK9 and IDOL was thought to be distinct, yet complementary;^16^ however, our data with RINGPep2 suggests that these two pathways are linked *via* a sterol-dependent feedback loop, whereby the inhibition of IDOL activity leads to an inhibition of PCSK9 transcription *via* inactivation of SREBP processing. We also observed the downregulation of both *LXR* genes (*LXRα* and *LXRβ*) in RINGPep2-treated cells. This unexpected finding may be occurring *via* inhibition of HNF-4α transcription factor by the increased uptake of linoleic acid (found in LDL molecules),^38^ arising from the increase in LDLR protein levels (Fig. 3f). The observed downregulation of LXRs upon inhibition of IDOL homodimerization has the wider implication of decreasing cellular efflux of cholesterol, as well as the potential to reduce IDOL protein levels. Taken together, this data suggests that inhibition of IDOL homodimerization has a wider impact beyond simply increasing LDLR levels; *de novo* cholesterol biosynthesis is downregulated and LDLR degradation by PCSK9 is also downregulated. While additional work is needed to probe and validate the above pathways, our data demonstrates the potential for using RINGPep2 as tool for this process, as well as the starting point for the development of therapeutics targeting IDOL homodimerization. This work further demonstrates the potential of cyclic peptides for targeting some of the most challenging target classes.

## Material and methods

### Construction of the IDOL RTHS

The RTHS used in this study was constructed as previously detailed.^24^ Briefly, IDOL was cloned into the multiple cloning site of pTHCP16 *via* the BamHI and SacI restriction endonuclease sites. This plasmid was integrated on to the HK022 site on the chromosome of *SNS118* (ref. 23) using the CRIM system.^39^ Formation of a functional repressor upon induction of the 434-IDOL fusion protein was assessed by drop spotting and ONPG assays as previously detailed.^21^,^24^

### SICLOPPS screening for IDOL dimerization inhibitors

Electrocompetent IDOL RTHS cells were prepared and transformed with a CX_5_ SICLOPPS plasmid library (constructed as previously detailed).^28^ Transformation efficiency was assessed by plating 10-fold serial dilutions of the recovery solution on LB agar supplemented with chloramphenicol (35 μg mL^–1^). Transformation efficiency was 7 × 10^6^, sufficient for 2-fold coverage of the 3.2 × 10^6^ member library. Transformants were plated onto minimal media agar plates supplemented with carbenicillin (50 μg mL^–1^), spectinomycin (25 μg mL^–1^), kanamycin (25 μg mL^–1^), 3-AT (2.5 mM), IPTG (25 μM), l-arabinose (6.5 μM) and chloramphenicol (35 μg mL^–1^). The plates were incubated for 2 days at 37 °C, until individual colonies were visible.

Surviving colonies were picked, grown overnight in LB supplemented with chloramphenicol (35 μg mL^–1^), and assessed for IDOL inhibition by drop-spotting 10-fold serial dilutions onto minimal media plates (supplemented with antibiotics and 3-AT as above), with and without IPTG and/or arabinose. Plasmids from strains displaying a significant growth advantage in the presence of IPTG and arabinose were isolated and transformed into a RTHS that replaced IDOL with an unrelated protein (C-terminal binding protein (CtBP))^20^ but was otherwise identical. Plasmids encoding cyclic peptides that resulted in a growth advantage in the CtBP RTHS were discarded as non-specific. The remaining plasmids were ranked for activity by drop-spotting on minimal media plates supplemented as above except with higher levels of 3-AT (5 mM) and kanamycin (50 μg mL^–1^). The identity of the variable region of the SICLOPPS plasmid encoding the most active cyclic peptides were deconvoluted by DNA sequencing.

### Synthesis of cyclic peptides

All peptides were synthesized by solid phase peptide synthesis (SPPS) on pre-loaded Wang resin. Synthesis of peptides containing cysteine was carried out with Fmoc-Cys(StBu)-OH. Peptides were cleaved from resin and acid labile protecting groups removed with TFA : TIS : H_2_O (95 : 2.5 : 2.5). The crude linear peptides were cyclized with HOBt (8 eq.) and EDC (4 eq.) in DMF (1 mg mL^–1^). The reaction mixtures were stirred for 24 h and concentrated *in vacuo*. The resulting peptide mixtures were purified by reverse-phase HPLC on a C18 column (Waters). The StBu protecting groups were removed from Cys containing peptides with DTT (20 eq.) in DMF:0.1 M NH_4_(CO_3_)_2_ (1 : 1), the reaction was stirred for 2 h at RT. Peptides were purified by HPLC and characterized by high resolution mass spectrometry.

### Synthesis of TAT-tagged cyclic peptides

Cyclic peptides were synthesized as above, except Fmoc-Cys(Trt)-OH was used and prior to cyclization, the Cys residue was protected with 2-aldrithiol (2 eq.) in DMF (1 mg mL^–1^). The reaction was stirred for 24 h under argon at RT, and concentrated *in vacuo*. The resulting solution was added dropwise to 50 mL cold diethyl ether, resulting in precipitation of the cyclic peptide, which was purified by HPLC as above. The aldrithiol-protected cyclic peptides (1 eq.) were dissolved in DMF (1 mg mL^–1^) and CGRKKRRQRRRPPQ (Tat-tag, 2 eq.) was added to the reaction dropwise. The reaction was stirred in an inert atmosphere for 24 h, followed by concentration *in vacuo*. The crude product was purified by HPLC as above and the product was characterized by mass spectrometry.

### Expression and purification of IDOL

Full length *IDOL* (1–445) was cloned with a TEV cleavable N-terminal 6xHis tag and IDOL FERM domains (M1-Q334, M1-A273 and E183-A273) were cloned with a TEV cleavable N-terminal HN6-lysine-tag into pFastbac1 for baculovirus expression. Recombinant baculovirus was generated using the Bac-to-Bac system. *Spodoptera frugiperda* Sf21 cells cultured in Sf900II SFM media at 27 °C were infected with baculovirus (MOI = 2) at a cell density of 2.5–3.0 × 10^6^ cells mL^–1^ and harvested after 72 h. Full length IDOL purified by batch mode affinity purification overnight incubation at 4 °C (50 mM Tris pH 8.2, 500 mM NaCl, 1% glycerol, 25 mM imidazole, 1 mM TCEP) followed immediately by a ion exchange purification (20 mM Tris pH 7.8, 75 mM NaCl, 10% glycerol, 1 mM TCEP) and run on size exclusion chromotography (20 mM Tris, pH 7.8, 200 mM NaCl, 10% glycerol, 1 mM TCEP). IDOL FERM domains were purified by batch mode affinity purification overnight incubation at 4 °C (20 mM K_2_HPO_4_ pH 7.8, 500 mM KCl, 10% glycerol, 25 mM imidazole, 1 mM TCEP). Protein was cleaved with TEV protease overnight at 4 °C dialysed in ion exchange buffer (50 mM MES pH 6.8, 100 mM NaCl, 10% glycerol, 1 mM TCEP). Cleaved IDOL FERM was further purified by ion exchange and size exclusion chromatography (50 mM MES pH 6.8, 500 mM NaCl, 10% glycerol, 1 mM TCEP).

### Expression and purification of ^15^N labelled IDOL RING domain

^15^N labelled IDOL RING domain (G356-I445) was expressed as TEV cleavable N-terminal HN6-Lysine-Tag in a pET vector for *E. coli* expression. *E. coli* was grown at 37 °C in Terrific Broth till an OD_600_ of 0.6 was reached. Cells were then pelleted and washed in cold PBS, cells were pelleted and resuspended in ^15^NH_4_Cl supplemented minimal media and grown at 16 °C overnight. ^15^N labelled RING domain was purified by affinity chromatography (50 mM Tris pH 8.0, 300 mM NaCl, 10% glycerol, 25 mM imidazole, 1 mM TCEP) followed by size exclusion chromatography (50 mM Tris pH 8.0, 150 mM NaCl, 0.5 mM TCEP). When required TEV cleavage was conducted at 4 °C overnight.

### Expression and purification of ^15^N labelled IDOL FERM domain

^15^N labelled IDOL FERM domain was expressed in BL21 *E. coli*. Cells were grown at 37 °C in Terrific Broth till an OD_600_ of 0.6 was reached. Cells were then pelleted and washed twice in cold PBS (500 mL), cells were pelleted and resuspended in ^15^NH_4_Cl supplemented minimal media and grown at 18 °C for 90 minutes prior to induction with IPTG (1 mM) and overnight growth. Cells were pelleted, harvested and lysed in buffer (20 mM Na_3_PO_4_ pH 7.8, 500 mM NaCl, 10% glycerol, 25 mM imidazole, 1 mM TCEP). ^15^N labelled FERM domain was purified by affinity chromatography using a HisTrap column, followed by size exclusion chromatography (50 mM Na_3_PO_4_ pH 7.8, 200 mM NaCl, 3 mM TCEP). The protein was further purified by chromatography on a Resource S column (50 mM Na_3_PO_4_ pH 6.8, 10 mM NaCl, 10% glycerol, 1 mM TCEP) eluting with a NaCl 1 M concentration gradient.

### Surface plasmon resonance (SPR)

All measurements were performed on a Biacore S200 (GE healthcare) using running buffer 10 mM HEPES, 300 mM NaCl, 1 mM TCEP, 0.05% Tween 20 and 4% DMSO at pH 8.0. In brief, 6× His-tagged, full-length IDOL was immobilized on a NTA-chip (Xantec), to a final level of ∼6000 RU. The chip was pre-loaded with Ni^2+^ to facilitate efficient capture of His-tagged protein onto the chip. The activity of the immobilized protein was confirmed using a reference compound.

### Two-dimensional protein NMR

^15^N-labeled RING and FERM domains were prepared for NMR experiments by buffer exchange using Amicon Ultra-4 Centrifugal Filter Units with 3 kDa molecular weight cut-off membranes. Buffer conditions for the NMR studies were 50 mM TRIS-d11 pH 7.5, 200 mM NaCl, 3 mM TCEP, 10% (v/v) D_2_O for the RING domain and 25 mM TRIS-d11, 25 mM NaPi, pH 7.5, 300 mM NaCl, 3 mM TCEP, 10% (v/v) D_2_O for the FERM domain. The protein concentrations were 80 μM and 70 μM, for the RING and FERM domain respectively. Two-dimensional NMR SOFAST-HMQC titration experiments were carried out on a Bruker 600 MHz spectrometer equipped with a cryoprobe. The NMR spectra were acquired at 293 K applying 192 and 96 scans for the FERM and the RING domain, respectively. ^1^H and ^15^N backbone assignments for the RING domain were adapted from BioMagResBank database (accession code 17550).^15^

### Microscale thermophoresis (MST)

MST was performed on a Monilith NT.115 system (Nano Temper Technologies, DE). 6× His IDOL was labelled with NT-647-NHS dye (Nano Temper Technologies, DE) as per manufacturer's instruction and used at a final concentration of 50 nM throughout the assays. Peptides were subject to dilution into assay buffer (10 mM HEPES, 300 mM NaCl, 1 mM TCEP, 0.05% TWEEN-20, pH 8.0) with a final assay concentration of 5% DMSO. Solutions containing peptide and labelled IDOL were loaded in to premium capillaries (Nano Temper Technologies, DE) for measurement.

### IDOL autoubiquitination assay

IDOL autoubiquitination was determined *in vitro*. 30 nM full length IDOL was incubated with inhibitor for 30 minutes at room temperature prior to the addition of 50 nM UBE1 (Ubiquigent, UK), 360 nM UBE2D2 (Ubiquigent, UK), 2 mM ATP (in 50 mM Tris, pH 8.0, 5 mM MgCl_2_, 100 mM NaCl), final volume 30 μL. The reaction was incubated at room temperature for three hours prior to the addition of 30 μL 2× Laemmli buffer. Ubiquitination was then detected *via* Western immunoblotting utilising anti-ubiquitin antibody (1 : 1000 dilution, MAB1510, Merk Millipore).

### Cell culture

HepG2 cells were purchased from ATCC and maintained in DMEM containing 10% foetal bovine serum (FBS). The HepG2 IDOL KO cell line was maintained in MEM containing 10% FBS, 1 mM sodium pyruvate and 0.1 mM non-essential amino acids (NEAA). All cell culture reagents were purchased from Life Technologies, unless otherwise stated.

### Generation of ΔIDOL HepG2 cells

An IDOL HepG2 knockout cell line (ΔIDOL HepG2 cells) was generated using CRISPR/Cas9 technology, as follows; a 41 bp deletion was generated in exon 4 by using dual guide approach. Two sgRNA were sub cloned into a vector expressing Cas9-T2A-GFP under the control of the CMV promotor, the sgRNAs were under the control of the U6 promotor. The guide sequences used were AGGGCAGAAACTGCTCAT (sgRNA1) and TGGAAAACTATGGCATAGAA (sgRNA2). The cells were transfected with plasmids expressing Cas9-T2A-GFP and sgRNA using the Neon Transfection system from Thermofisher. Cells were single cell sorted 48 h after transfection on GFP expression. Single cell clones were ultimately analyzed using next-generation sequencing. One clone with 41 bp deletion on 2 alleles and a 13 bp deletion on the third allele were further expanded and characterized. The cell line was maintained in Modified eagle's medium (MEM) supplemented with 1 mM Na pyruvate, 0.1 mM non-essential amino acids (NEAA) and 10% FBS.

### Western immunoblotting

Cells were plated in 60 mm plates at a density of 1 × 10^6^ cells per plate and incubated overnight (37 °C, 5% CO_2_). Cells were then treated with inhibitor and incubated for 72 h (37 °C, 5% CO_2_). Treated cells were collected following trypsinization, the resultant cell pellet was resuspended in PBS (2 mL) and centrifuged (1000 rpm, 4 min) and supernatant discarded. This wash step was repeated twice. Cells were lyzed by incubation with 30 μL RIPA buffer containing protease inhibitors on ice for 15 min, followed by sonication in an ice cold water bath (6 cycles of 30 s on 30 s off repeated twice). The cell debris was pelleted by centrifugation (10000 rpm, 10 min, 4 °C) and supernatant containing soluble protein collected. 30 μg protein was added to 2× Laemmli buffer and the proteins denatured at 90 °C for 10 min. Whole cell proteins were separated on a 12% (v/v) SDS-PAGE gel under denaturing conditions (150 V, 40–60 min) and transferred to a PVDF membrane (Invitrogen; 250 mA, 2 h). The membrane was blocked with 5% (v/v) non-fat powdered milk and 0.1% Tween-20 (v/v) in PBS and subject to immunoblot analysis. Rabbit anti-human LDLR (EP1553Y, Abcam) or Anti-MYLIP (IDOL) (ab74562, Abcam) antibody was diluted 1 : 5000 in 5% milk-PBS/Tween and incubated with the membrane overnight at 4 °C with rolling. The membrane was washed in PBS/Tween and incubated with anti-rabbit horseradish peroxidase (HRP) conjugated secondary antibody (7074, Cell Signalling), diluted 1 : 10000 in 5% milk-PBS/Tween for 1 h at room temperature. Bound immunocomplexes were detected using ECL prime Western blot detection reagent (RPN2232, GE Healthcare) and visualized on a Bio-Rad ChemiDOC.

### RNA isolation and qPCR

Following treatment, cells were collected by trypsinization and transferred to a 15 mL falcon tube and centrifuged (1000 rpm, 4 min). The supernatant was discarded and the pellet was washed twice in 2 mL PBS. The pellet was resuspended in the appropriate volume of BL buffer containing thioglycerol (Reliaprep RNA miniprep kit, Promega, UK). The cells were homogenised by vortexing and passed through a 200 μL pipette tip 5 times. The resultant extracted RNA was purified using the Reliaprep RNA miniprep columns (Promega), according to the manufacturer's instructions. RNA was eluted with DEPC (diethylpyrocarbonate) treated deionised water (1% v/v) to avoid the introduction of RNase and stored at –80 °C. Reverse transcription (RT) reactions were carried out using the GoScript cDNA synthesis kit (Promega) according to the manufacturer's instructions. qPCR was carried out using TaqMan gene expression assays (ThermoFisher Scientific), specific to the gene of interest. All samples were run in triplicate, alongside a no template control and two housekeeper genes, typically β-actin and 18S.

### Cell viability assay

Cell viability was assessed using the CellTiter-Glo 2.0 assay (Promega) according to the manufacturer's instructions. Briefly, cells were plated at a density of 3.3 × 10^4^ cells per well in a 96 well plate and incubated overnight (37 °C, 5% CO_2_). Cells were treated with increasing concentrations of inhibitor and incubated in lipoprotein depleted serum for 72 h (37 °C, 5% CO_2_). The plate was equilibrated to room temperature for 30 min prior to the addition an equal volume of CellTiter-Glo 2.0 (Promega) reagent to media. The contents were mixed for 2 min on an orbital shaker to induce cell lysis and the plate incubated at room temperature for 10 min prior to the detection of a luminescent signal.

## Data availability statement

Data underlying these experiments are available from the University of Southampton data repository at https://doi.org/10.5258/SOTON/D0566.

## Conflicts of interest

E. K. L. and A. T. are co-inventors on UK patent application “Cyclic Peptide inhibitors of IDOL homodimerization”.

## Supplementary Material

Supplementary information
