# Supplementary material for: Inhibition of low-density lipoprotein receptor degradation with a cyclic peptide that disrupts the homodimerization of IDOL E3 ubiquitin ligase
Source: Chem Sci. 2018 Jun 26;9(27):5957–66. doi: 10.1039/c8sc01186a (PMC6050537; doi:10.1039/c8sc01186a)

Electronic Supplementary Material (ESI) for Chemical Science.
This journal is © The Royal Society of Chemistry 2018

Supplementary Information

Inhibition of low-density lipoprotein receptor degradation with a cyclic peptide that disrupts the homodimerization of IDOL E3 ubiquitin ligase.

Eilidh K. Leitch,[1] Nagarajan Elumalai,[1] Maria Fridén-Saxin,[2] Göran Dahl,[3] Paul Wan,[3] Paul Clarkson,[4] Eric Valeur,[2] Garry Pairaudeau,[4] Helen Boyd,[5] Ali Tavassoli[1,6]*

[1]Chemistry, University of Southampton, Southampton, SO17 1RE, United Kingdom.

[2]Medicinal Chemistry, Cardiovascular and Metabolic Diseases, IMED Biotech Unit, AstraZeneca, Pepparedsleden 1, Mölndal, 43150, Sweden.

[3]Structure and Biophysics, Discovery Sciences, IMED Biotech Unit, AstraZeneca, Pepparedsleden 1, Mölndal, 43150, Sweden.

[4]AstraZeneca, Cambridge Science Park, 310 Milton Rd, Cambridge, CB4 0FZ, United Kingdom.

[5]Drug Safety and Metabolism, IMED Biotech Unit, AstraZeneca, Pepparedsleden 1, Mölndal, 43150, Sweden.

[6]Insititute for Life Sciences, University of Southampton, Southampton, SO17 1BJ, United Kingdom.

*e-mail: a.tavassoli@soton.ac.uk

Supplementary Figures

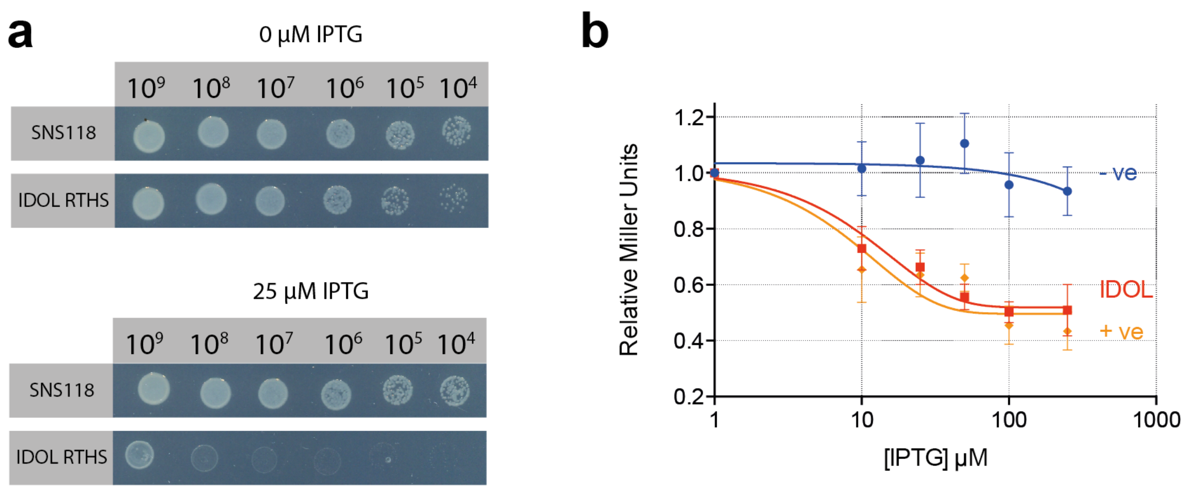

Supplemental Figure 1: Verification of IDOL homodimer PPI formation within RTHS. a) Assessing function of the IDOL RTHS by drop spotting 10-fold serial dilutions (2.5 L of ~ 10^n cells/mL). The blank *SNS118* strain is used as a negative control. In the absence of IPTG both strains grow fully (6 spots), however when grown with 25 µM IPTG (inducer of 434-IDOL) the formation of a functional 434 repressor in the IDOL RTHS prevents growth and survival of the IDOL RTHS strain (due to inhibition of reporter-gene expression), while the blank *SNS118* strain grows fully. b) ONPG assay of the IDOL RTHS. The data shows increasing IPTG concentrations (increasing expression of 434-IDOL) resulting in reduced activity of the *LacZ* reporter gene product (β-galactsidase), as quantified by monitoring the cleavage of ONPG. This further confirms the formation of a functional repressor via dimerization of IDOL in the IDOL RTHS.

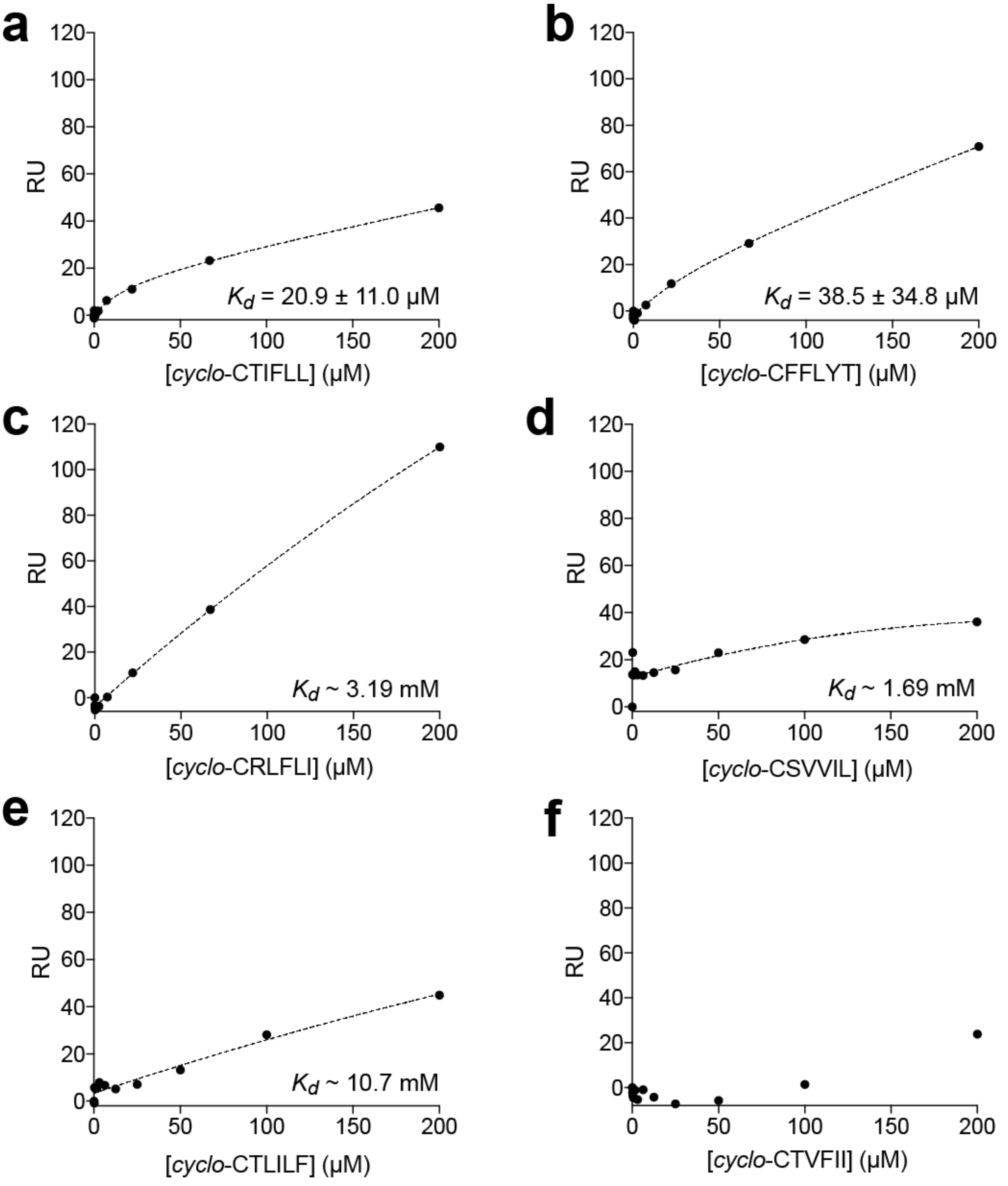

Supplementary Figure 2. Assessing IDOL binding of primary hits by SPR. Full length His-IDOL was immobilised and 6 lead peptides from the SICLOPPS screen were analyzed in a 12-point dilution series for binding to IDOL. Saturation of binding was not observed due to the hydrophobic nature of the peptides

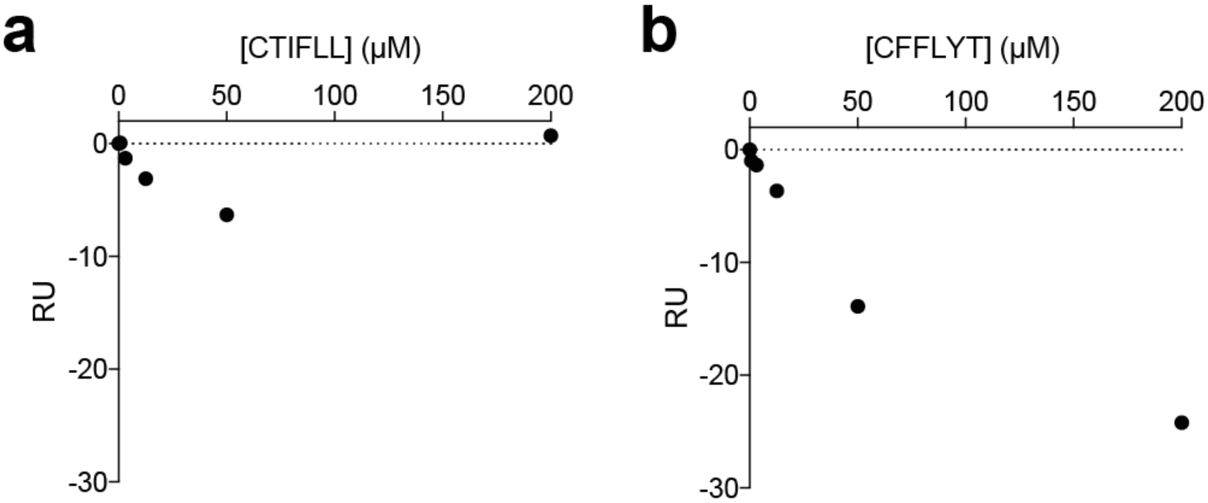

Supplementary Figure 3. SPR analysis of linear peptide derivatives against IDOL. Full length His-IDOL was immobilised and two linear peptide derivatives (a) linear CTIFLL and (b) linear CFFLYT were analysed in a 6-point dilution series for binding to IDOL.

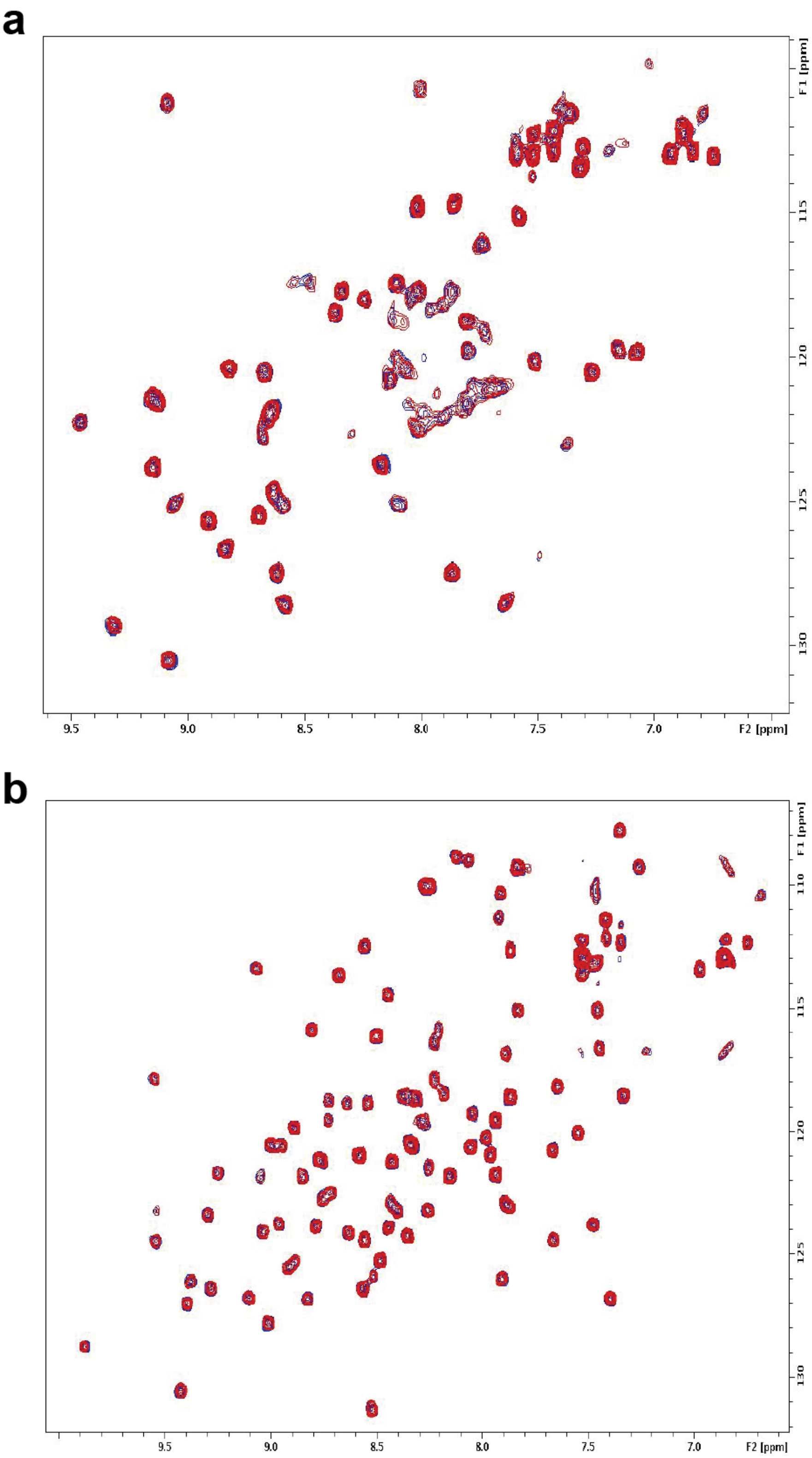

Supplementary Figure 4. ^{1}H, ^{15}N HSQC NMR chemical shift mapping of the IDOL RING and FERM domains with *cyclo*-CTIFLL. ^{1}H ^{15}N HSQC spectra of a) 80 μM ^{15}N-labelled RING and b) 70 μM ^{15}N-labelled FERM domains in the absence (blue) and presence (red) of *cyclo*-CTIFLL at 8 equivalents. No significant chemical shift changes were observed against either domain.

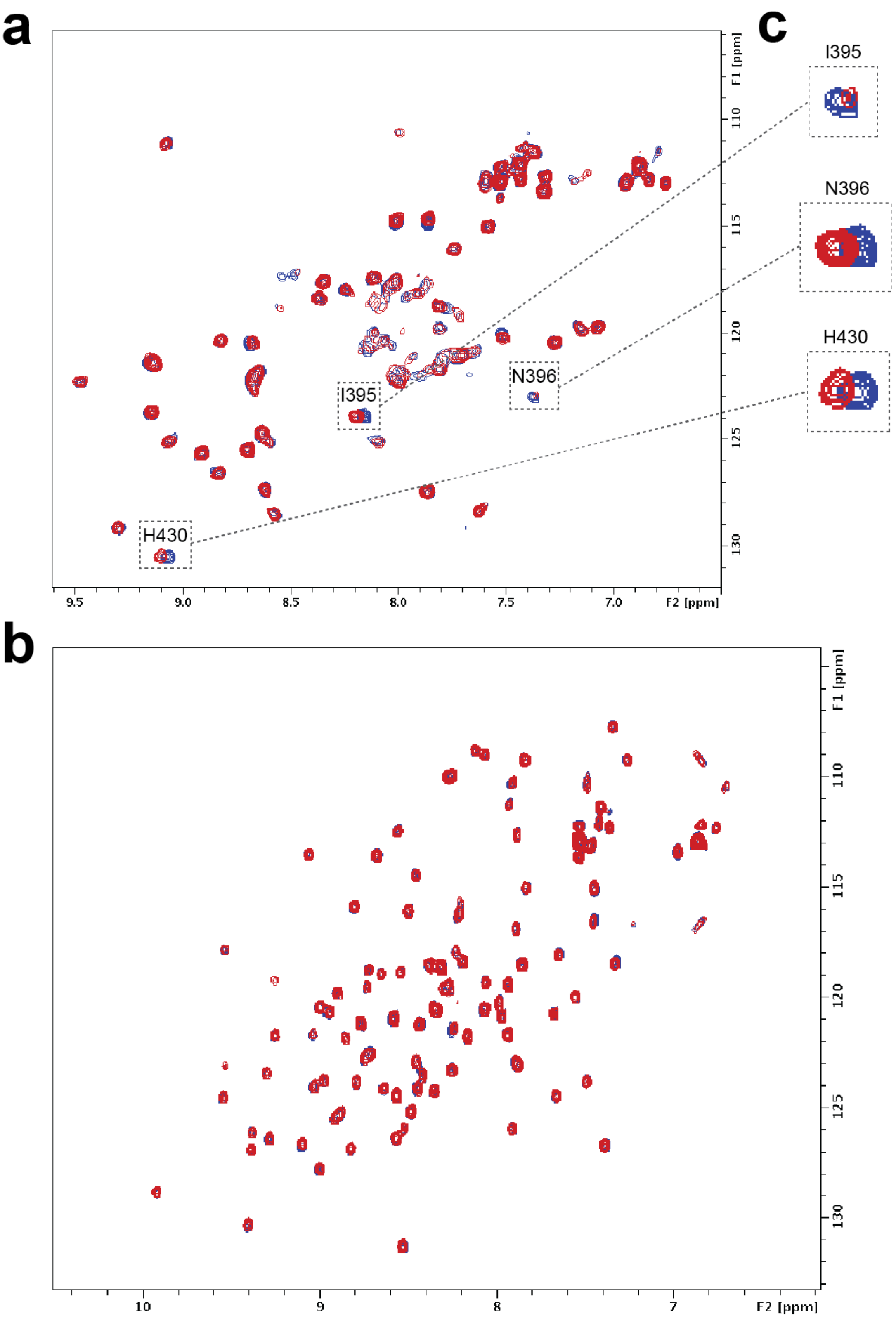

Supplementary Figure 5. ^{1}H, ^{15}N HSQC NMR chemical shift mapping of the IDOL RING and FERM domains with *cyclo*-CFFLYT. ^{1}H ^{15}N HSQC spectra of a) 80 µM ^{15}N-labelled IDOL RING domain and b) 70 µM ^{15}N-labelled IDOL FERM domain; in the absence (blue) and presence (red) of *cyclo*-CTIFLL at 8 equivalents. c) Close-up of the shifts observed for I395, N396, and H430.

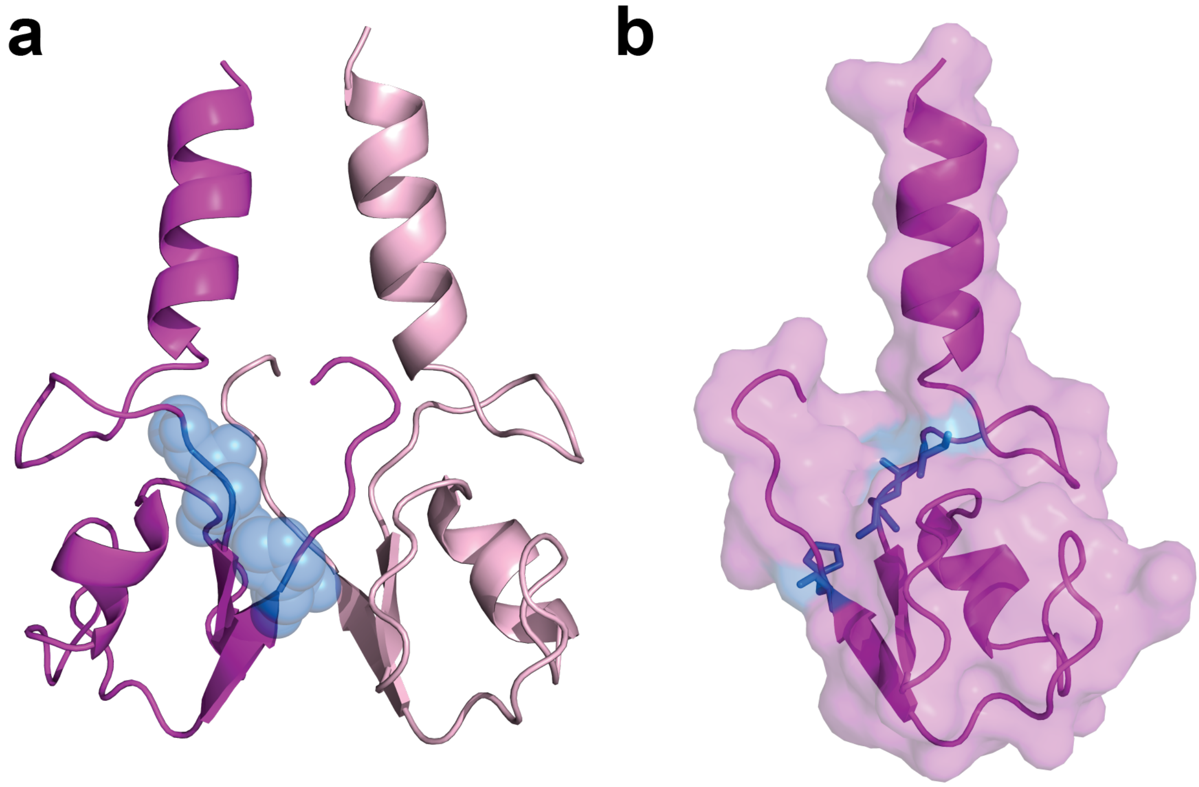

Supplementary Figure 6. IDOL residues perturbed by RINGPep1. a) the IDOL homodimer is show in cartoon form, with the residues perturbed by RINGPep1 by ^{1}H ^{15}N HSQC NMR shown in blue. b) the image in panel a is rotated on the y-axis by -90°. The homodimerization interface is shown, with the monomer closest to the viewer removed so that the residues affected by RINGPep1 are seen in relation to the homodimerization interface.

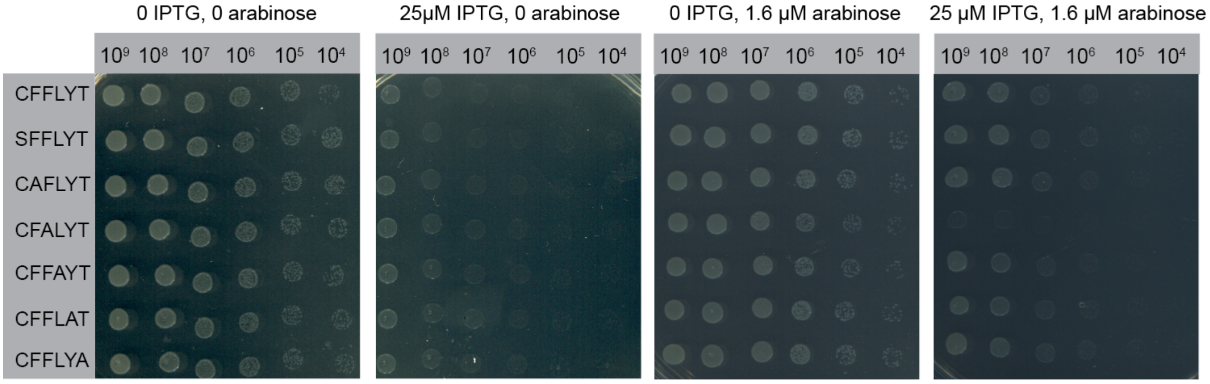

Supplementary Figure 7. Alanine scanning of RINGPep1 using the IDOL RTHS. SICLOPPS plasmids encoding alanine analogues of RINGPep1 were introduced into the IDOL RTHS and drop-spotted on minimal media selection plates. SFFLYT was used in place of AFFLYT to allow intein splicing and cyclic peptide production. The IDOL RTHS fully gros in the absence of IPTG or arabinose, and in all cases, growth is repressed by 25μM IPTG (encoding for 434-IDOL) indicating formation of a functional repressor. Growth of the IDOL RTHS is not affected by 1.6 μM arabinose (inducer of SICLOPPS) indicating no toxicity from the peptides. In the presence of 25 μM IPTG and 1.6 μM arabinose (both IDOL and SICLOPPS being produced) growth is partially restored via all cyclic peptides except for CFALYT (which does not grow). This indicates that the 2nd F in RINGPep1 is critical for disruption of IDOL homodimerization.

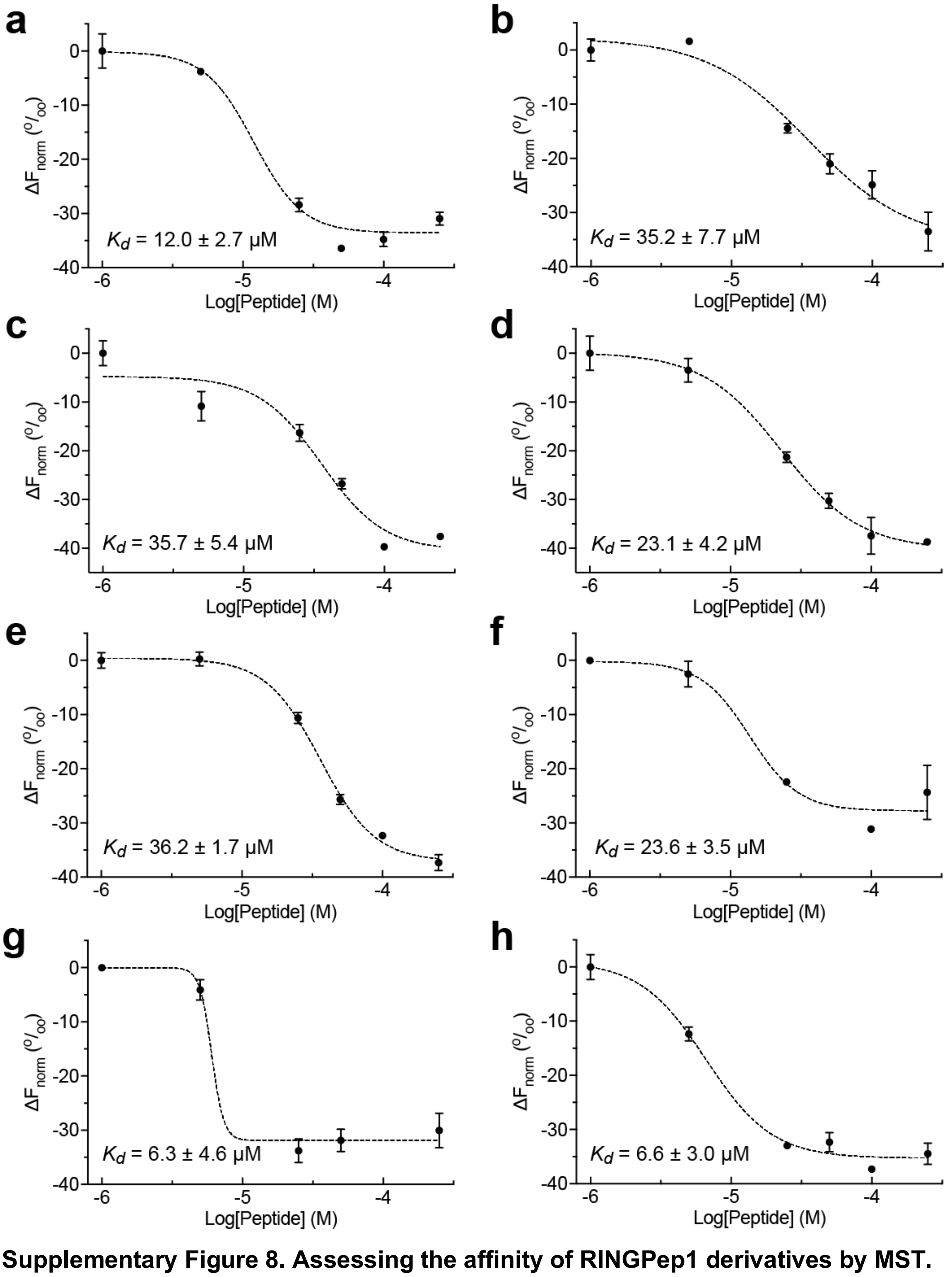

Supplementary Figure 8. Assessing the affinity of RINGPep1 derivatives by MST. Derivatives of *cyclo*-CFFLYT with non-natural L-phenylalanine analogues incorporated into position 3 of the cyclic peptide were synthesized and their binding to full length His-IDOL (50 nM) analysed by MST. The following non-natural derivative were used: a) 4-chloro-phenylalanine, b) 4-fluoro-phenylalanine, c) 4-nitro-phenylalanine, d) 4-methy-phenylalanine, e) 4-cyano-phenylalanine, f) homo-phenylalanine, g) 3-(1-naphthyl)-alanine h) 3-cyclohexyl-alanine. All data represented as mean ± SEM, n = 3.

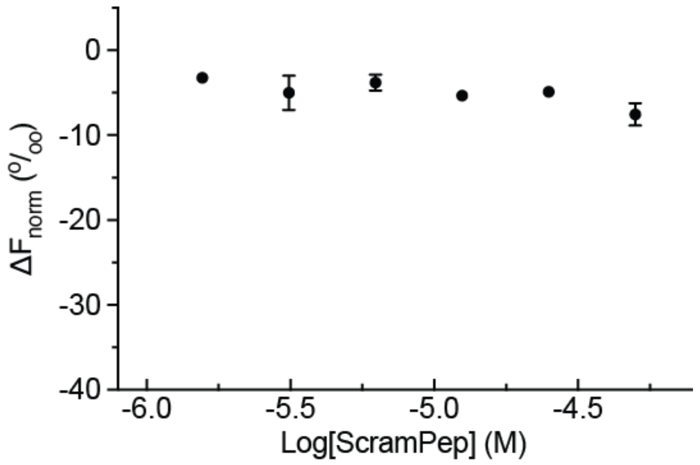

Supplementary Figure 9. Structure and binding analysis of cyclo-CYLFT[Cha] (ScramPep). The binding of ScramPep to full Length His-IDOL (44 nM) was assessed by MST. Data represented as mean ± SEM, n = 3.

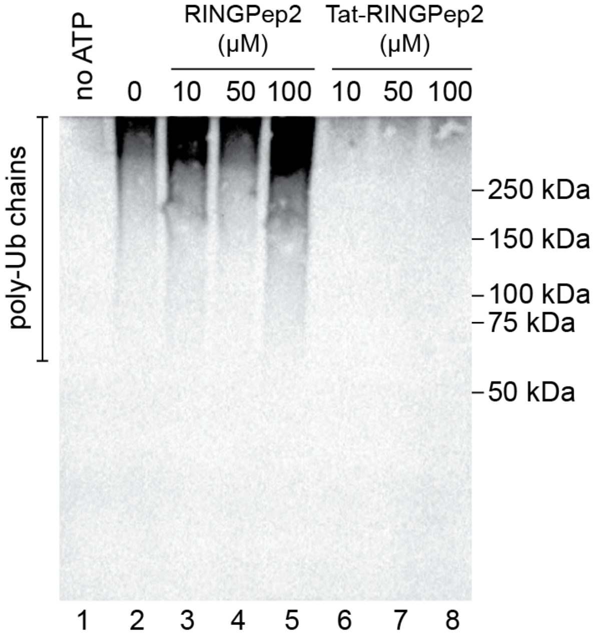

Supplementary Figure 10. The effect of RINGPep2 on IDOL autoubiquitination *in vitro*. RINGPep2 and Tat-RINGPep2 were incubated with IDOL and their effect on IDOL autoubiquitination assessed in an *in vitro* assay. A reaction lacking ATP was used as control.

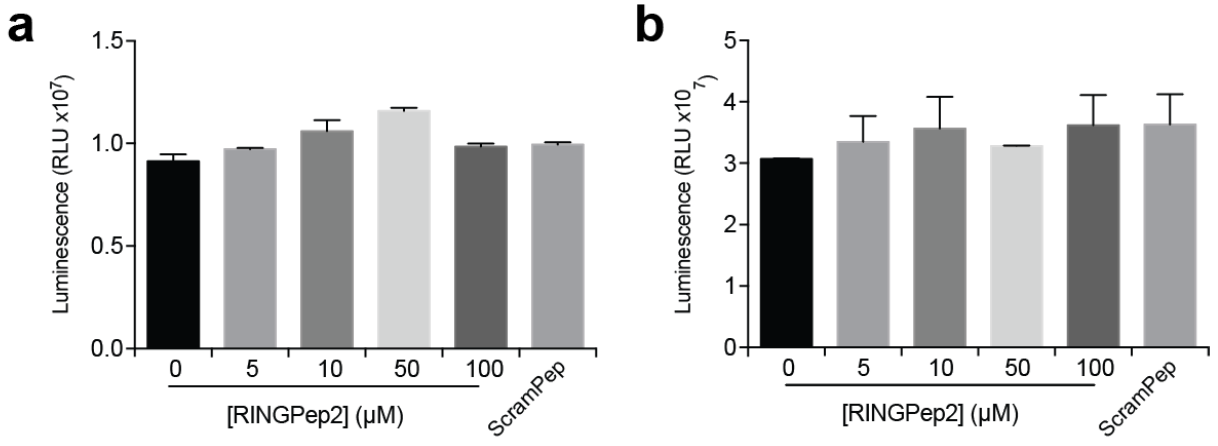

Supplementary Figure 11. Cellular viability following peptide treatment. The viability of (a) HepG2 and (b) ΔIDOL HepG2 cells was determined following treatment with increasing concentrations of TAT-RINGPep2 and TAT-ScramPep (100 μM). Data represented as mean ± SEM, n = 3.

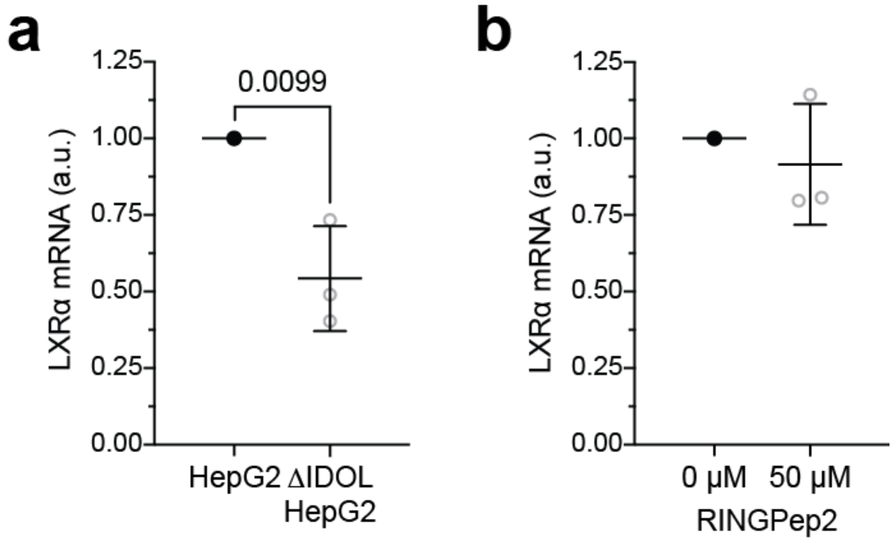

Supplementary Figure 12. The effect of IDOL and RINGPep2 on the transcription of LXRα. a) Comparing the expression of *LXRα* in HEPG2 cells with that in ΔIDOL HepG2 cells. b) ΔIDOL HepG2 cells were treated 0 μM or 50 μM RINGPep2 and of *LXRα* mRNA levels detected by qPCR. All data represented as mean ± SEM, n = 3.

Supplementary Materials and Methods

Peptide synthesis

cyclo-CTLILF

The peptide was synthesised using standard solid phase peptide synthesis techniques, yielding 1.6 mg (6%) as a white solid. LRMS (ESI$^+$): *m/z* 691.4 [M+H]$^+$, 714.4 [M+Na]$^+$, 100%; HRMS (ESI$^+$) $C_{34}H_{54}N_6O_7S$ found *m/z* 691.3854 [M+H]$^+$; calculated 691.3847 (-1.0 ppm error) and *m/z* 713.3674 [M+Na]$^+$; calculated 713.3667 (-0.9 ppm error). HPLC retention time 1.92 min.

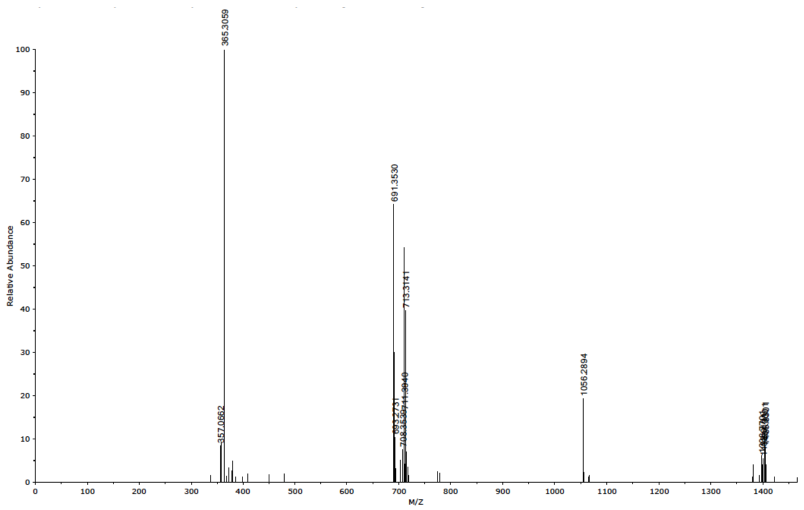

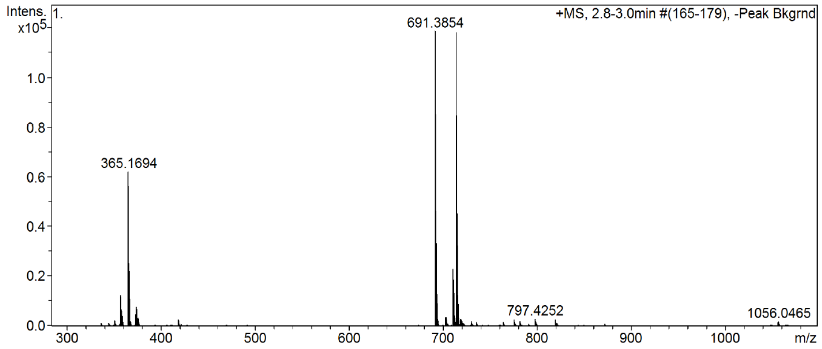

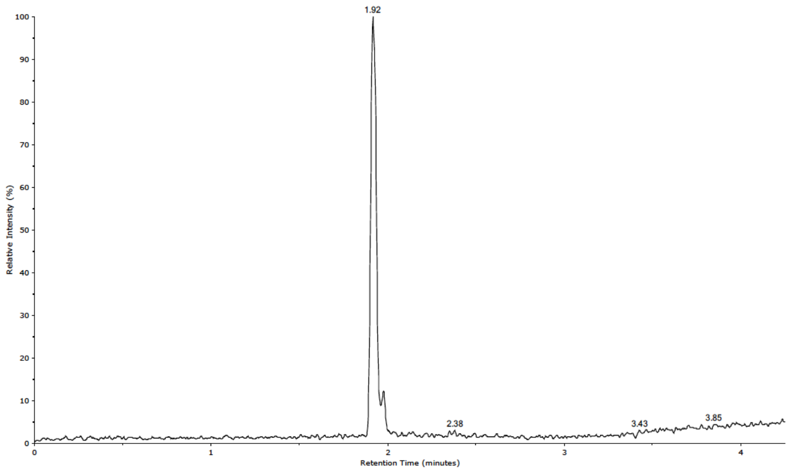

<u>*cyclo*-CTVFII</u>

The peptide was synthesised using standard solid phase peptide synthesis techniques, yielding 3.6 mg (25%) as a white solid. LRMS (ESI^+): *m/z* 677.4 $[M+H]^{+,}$ 100%; HRMS (ESI^+) $C_{33}H_{52}N_6O_7S$ found *m/z* 677.3700 $[M+H]^+$; calculated 677.3691 (-1.4 ppm error) and *m/z* 699.3513 $[M+Na]^+$; calculated 699.3510 (-0.4 ppm error). HPLC retention time 1.90 min.

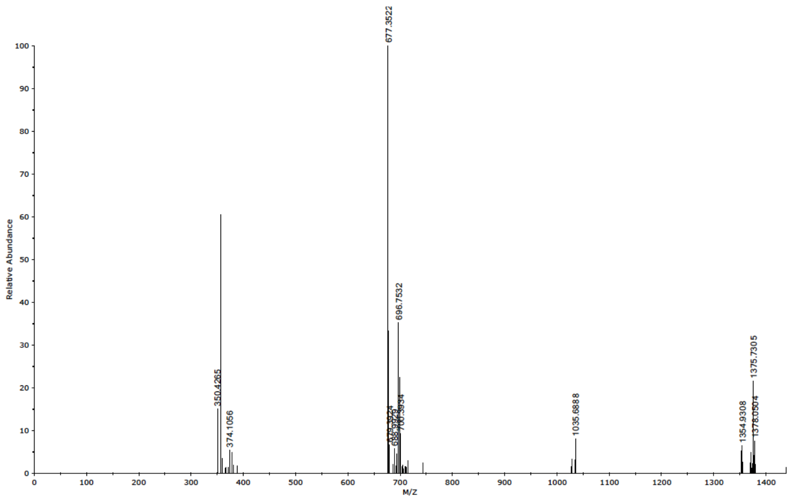

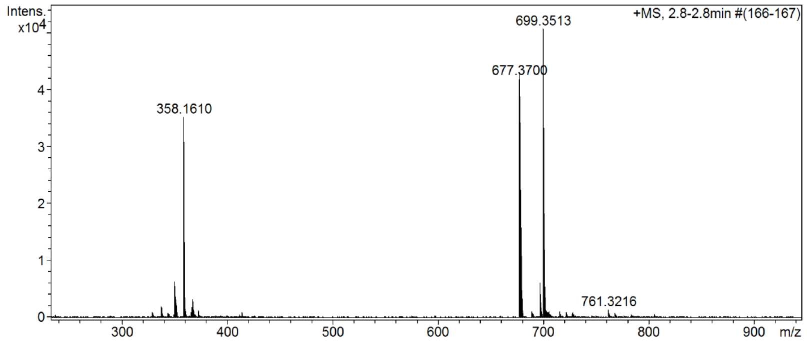

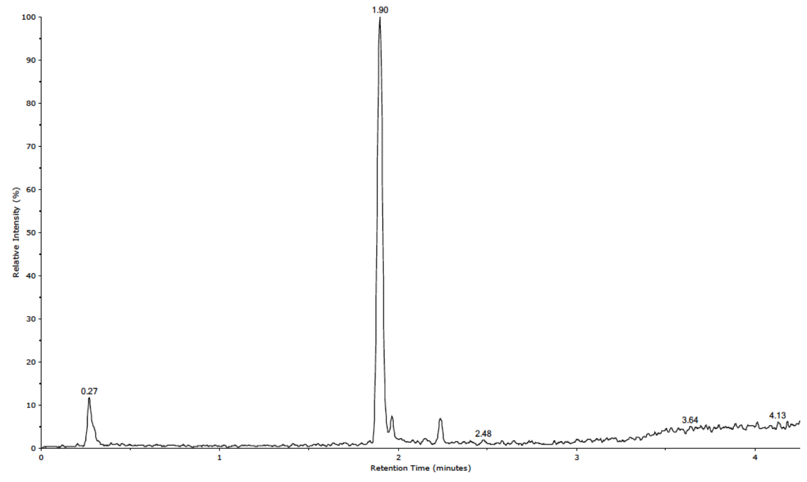

cyclo-CSVVIL

The peptide was synthesised using standard solid phase peptide synthesis techniques, yielding 8 mg (7% yield) as a white solid. LRMS (ESI$^+$): *m/z* 615.5 $[M+H]^+$ 100%, ; HRMS (ESI$^+$) for $C_{28}H_{51}N_6O_7S$ found *m/z* 615.3549 $[M+H]^+$; calculated 615.3534 and *m/z* 637.3366 $[M+Na]^+$; calculated 637.3357. HPLC retention time 2.06 (RP-HPLC, 5min, CH_3CN 20% to 100%).

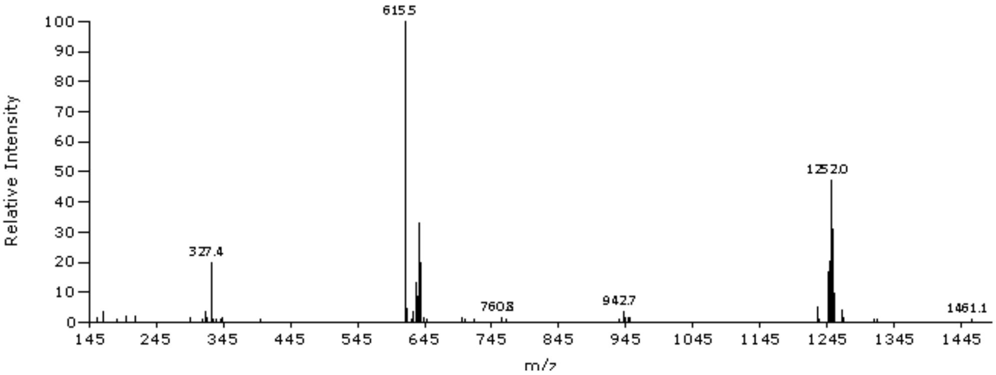

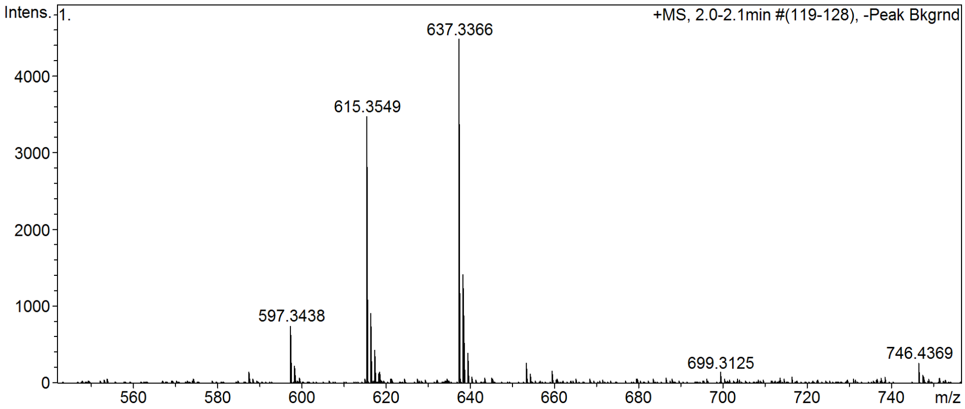

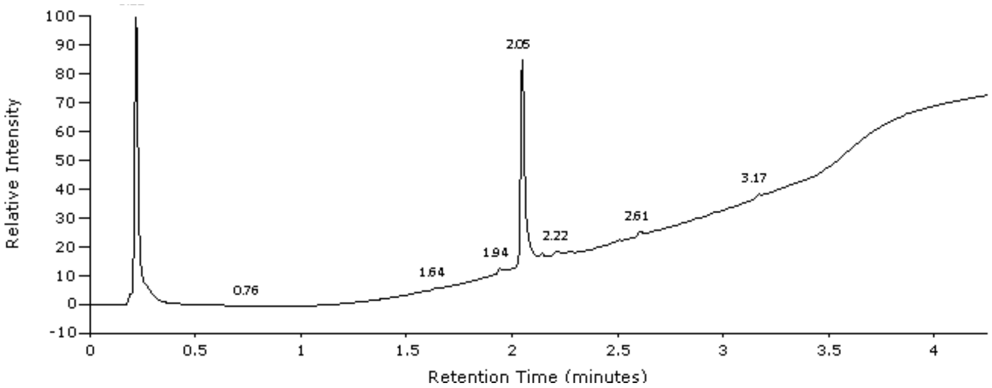

cyclo-CTIFLL

The peptide was synthesised using standard solid phase peptide synthesis techniques, yielding 22 mg (11.5%) as a white solid. LRMS (ESI$^+$): *m/z* 691.6, $[M+H]^+$; HRMS (ESI$^+$) for $C_{38}H_{62}N_6O_7S_2$ found *m/z* 691.3847 $[M+H]^+$; calculated 691.3847 and *m/z* 713.3663 $[M+Na]^+$; calculated 713.3667. HPLC retention time 4.60 min (RP-HPLC, 10min, CH_3CN 5% to 100%).

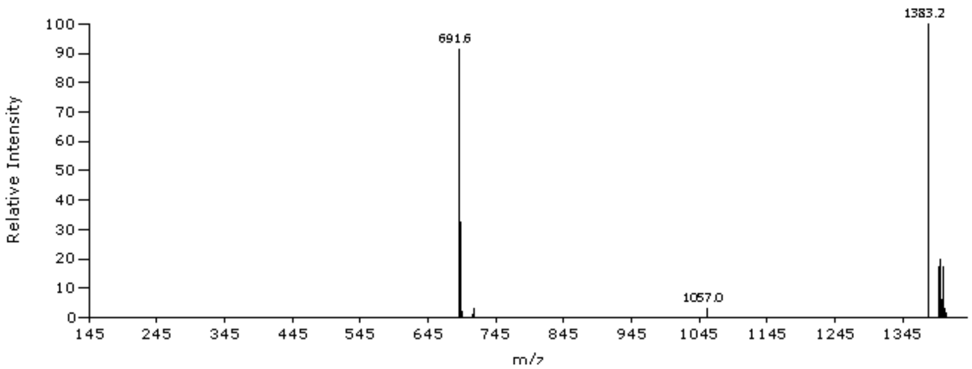

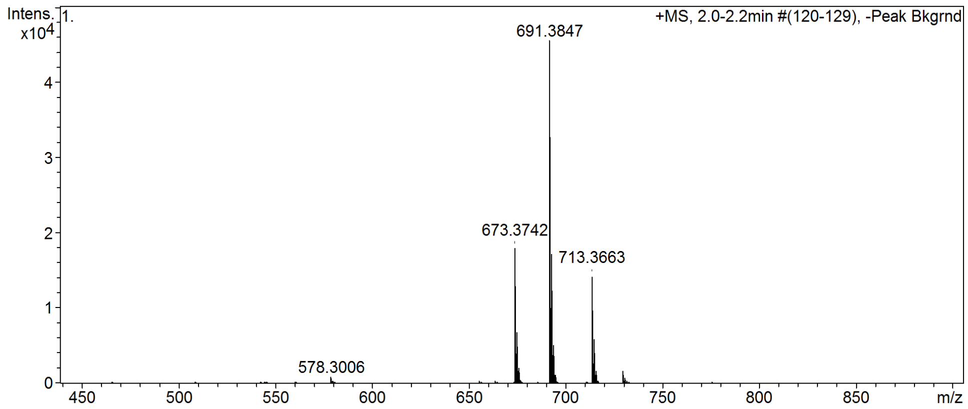

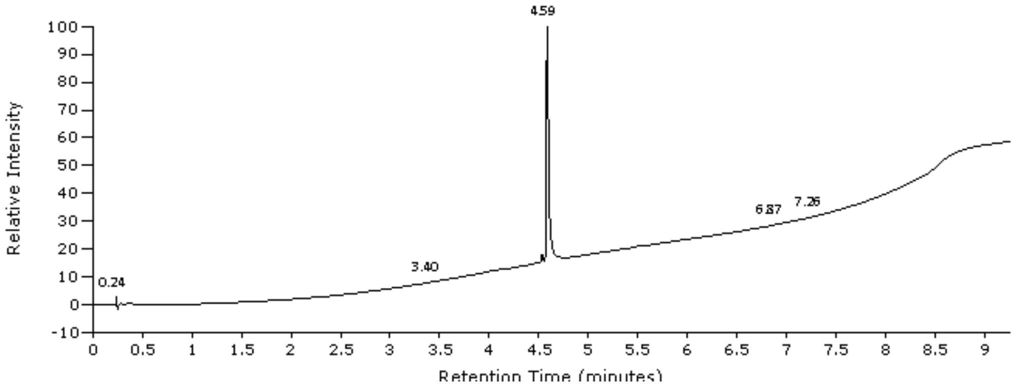

<u>*cyclo*-CRLFLI</u>

The peptide was synthesised using standard solid phase peptide synthesis techniques, yielding 22 mg (10.5% yield) as a white solid. LRMS (ESI$^+$): *m/z* 746.7 $[M+H]^+$, 100%; HRMS (ESI$^+$) for $C_{36}H_{60}N_9O_6S$ found *m/z* 746.4381 $[M+H]^+$; calculated 746.4382. HPLC retention time 1.8 min (RP-HPLC, 10min, CH_3CN 5% to 100%).

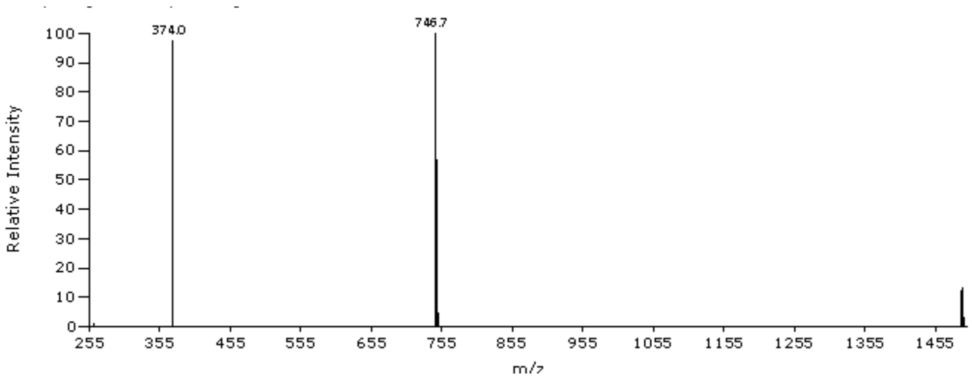

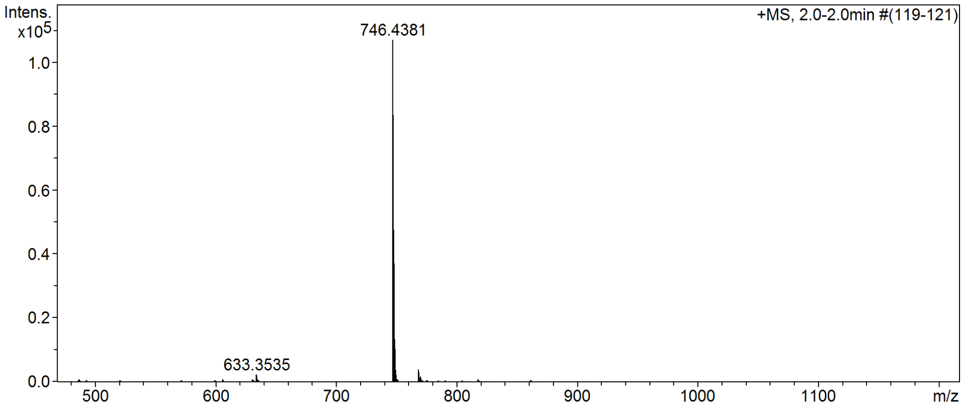

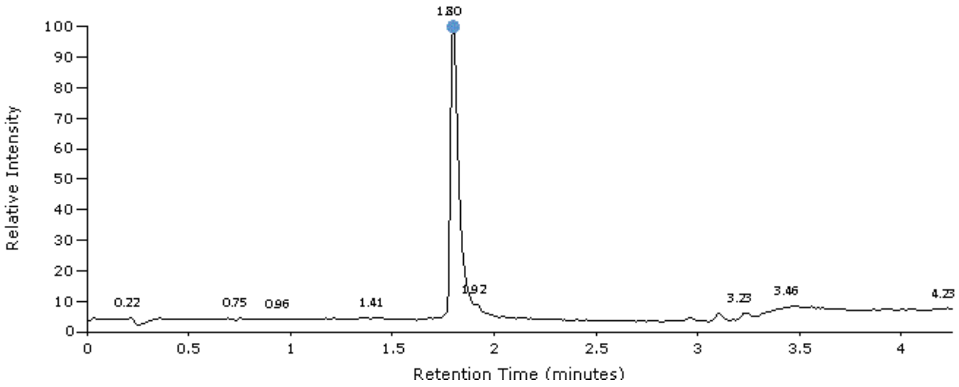

<u>*cyclo*-CFFLYT (RINGPep1)</u>

The peptide was synthesised using standard solid phase peptide synthesis techniques, yielding 32.5 mg (23.8%) as a white solid. LRMS (ESI$^+$): *m/z* 775.6, 100 % $[M+H]^+$; HRMS (ESI$^+$) for $C_{40}H_{50}N_6O_8S$ found *m/z* 775.3490 $[M+H]^+$; calculated 775.3484 (-0.8 ppm error). HPLC retention time 1.74 min.

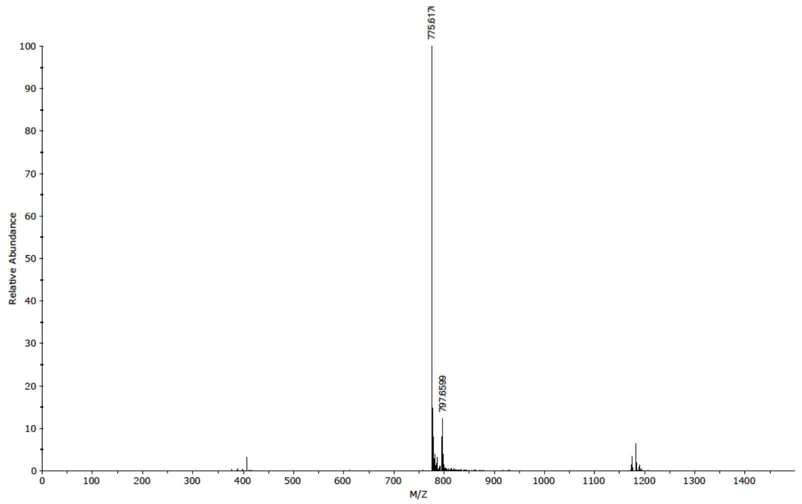

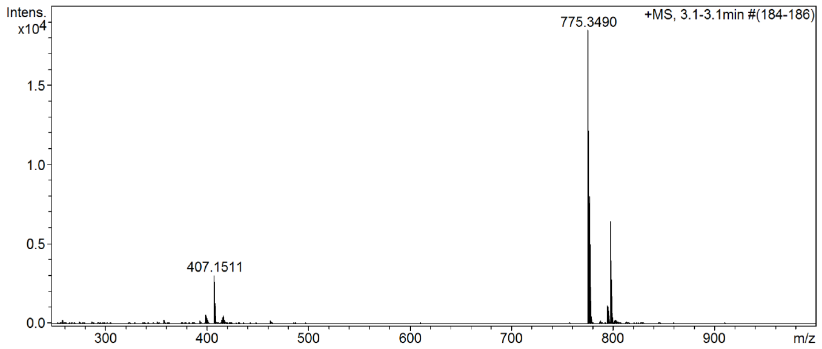

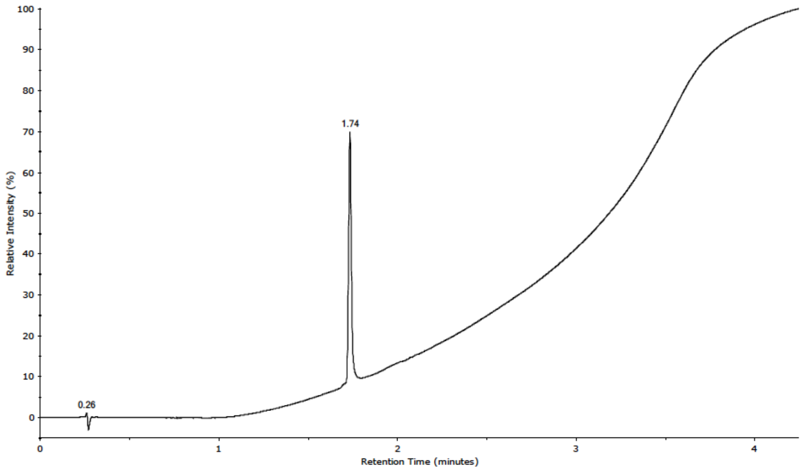

cyclo-CF[(4-Cl)F]LYT

The peptide was synthesised using standard solid phase peptide synthesis techniques, yielding 12.8 mg (6.3%) as a white solid. LRMS (ESI$^+$): *m/z* 809.5, 49.42% [M+H]$^+$, 809.5, 100% [M+H]$^+$; HRMS (ESI$^+$) for $C_{40}H_{49}ClN_6O_8S$ found *m/z* 809.3083 [M+H]$^+$; calculated 809.3094 (1.4 ppm error) and *m/z* 831.2906 [M+Na]+; calculated 831.2913 (0.9 ppm error). HPLC retention time 2.77 min.

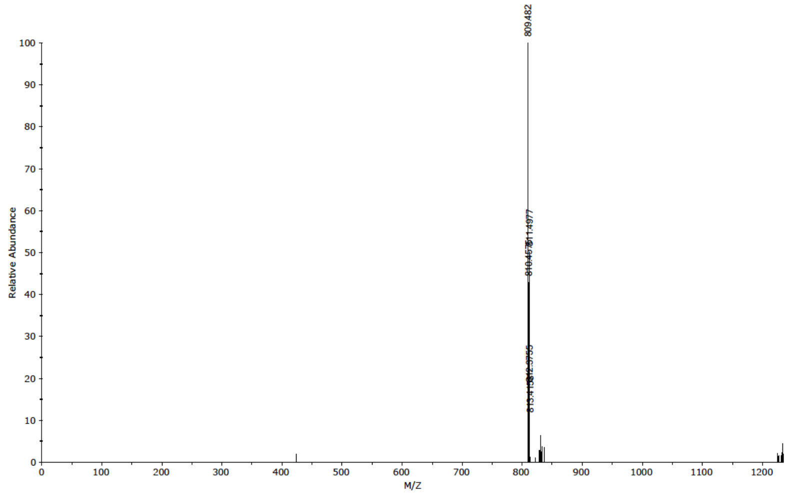

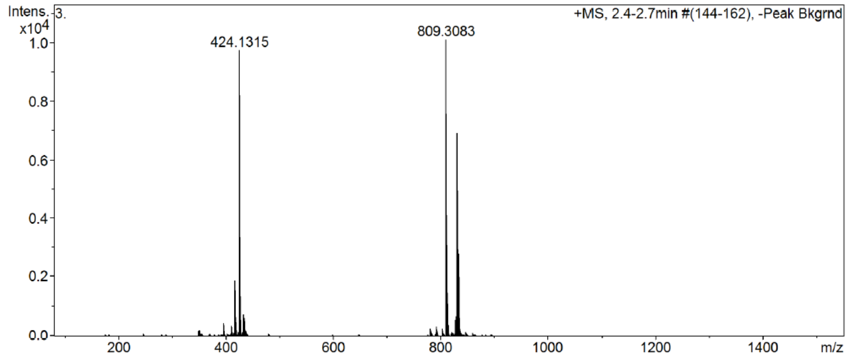

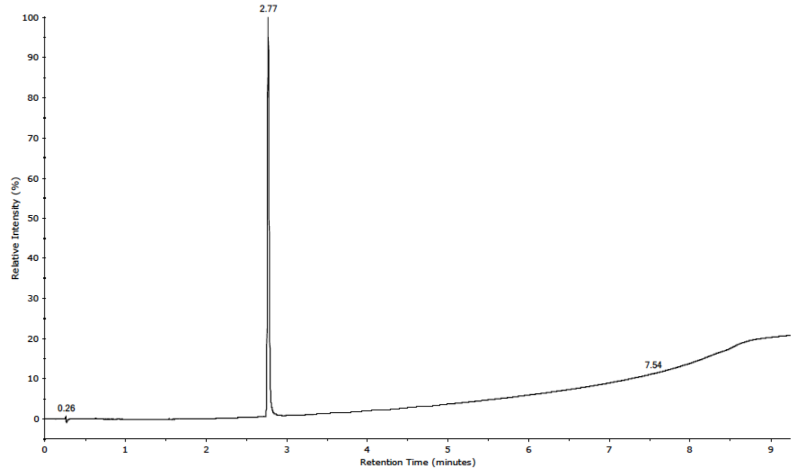

<u>*cyclo*-CF[(4-F)F]LYT</u>

The peptide was synthesised using standard solid phase peptide synthesis techniques, yielding 9.7 mg (4.9%) as a white solid. LRMS (ESI$^+$): *m/z* 793.5, $[M+H]^+$, 1586.7 $2[M+H]^+$, 100%; HRMS (ESI$^+$) for $C_{40}H_{49}FN_6O_8S$ found *m/z* 793.3391 $[M+H]^+$; calculated 793.3389 (-0.2 ppm error) and *m/z* 815.3208 $[M+Na]^+$; calculated 815.3209 (0.1 ppm error).HPLC retention time: 2.58 min.

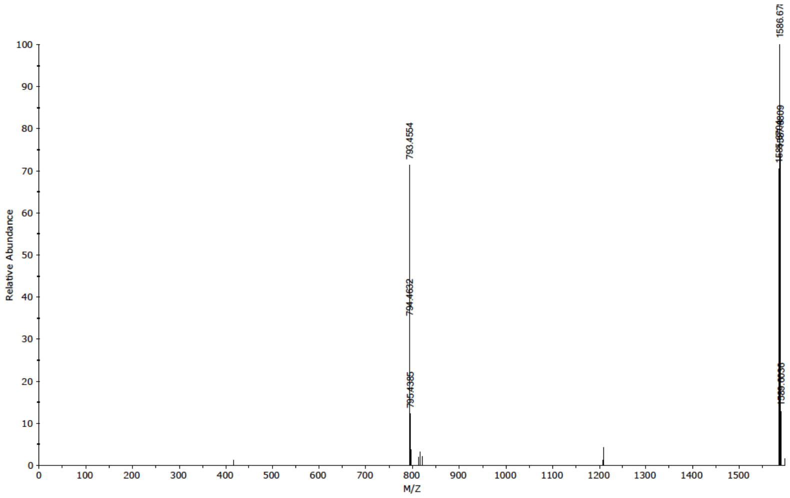

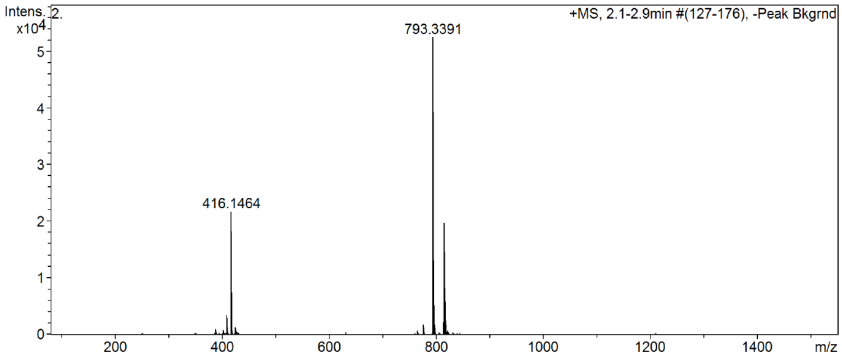

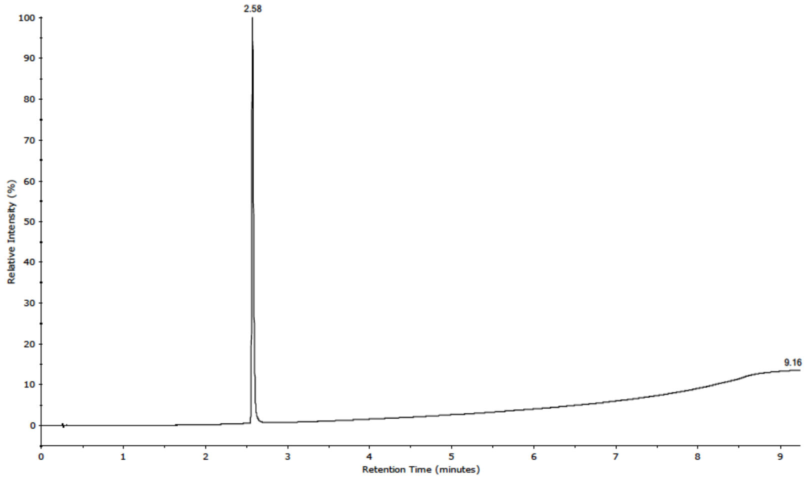

Cyclo-CF[(4-NO$_2$)F]LYT

The peptide was synthesised using standard solid phase peptide synthesis techniques, yielding 7.1 mg (3.5%) as a white solid. LRMS (ESI$^+$): *m/z* 820.4, 79.83% [M+H]$^+$, 820.4, 100% [M+H]$^+$; HRMS (ESI$^+$) for $C_{40}H_{49}N_7O_{10}S$ found *m/z* 820.3349 [M+H]$^+$; calculated 820.3334 (-1.8 ppm error) and *m/z* 842.3169 [M+Na]$^+$; calculated 842.3154 (-1.8 ppm error). HPLC retention time: 2.50 min.

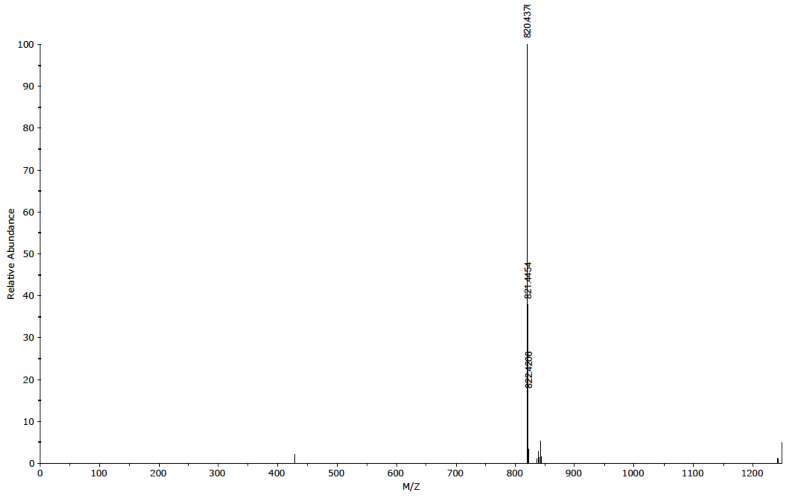

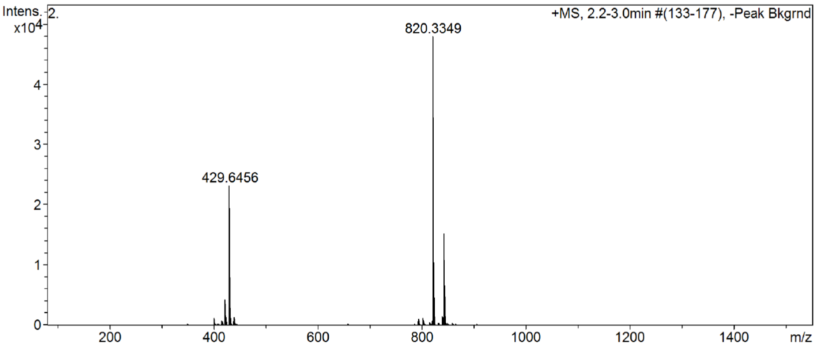

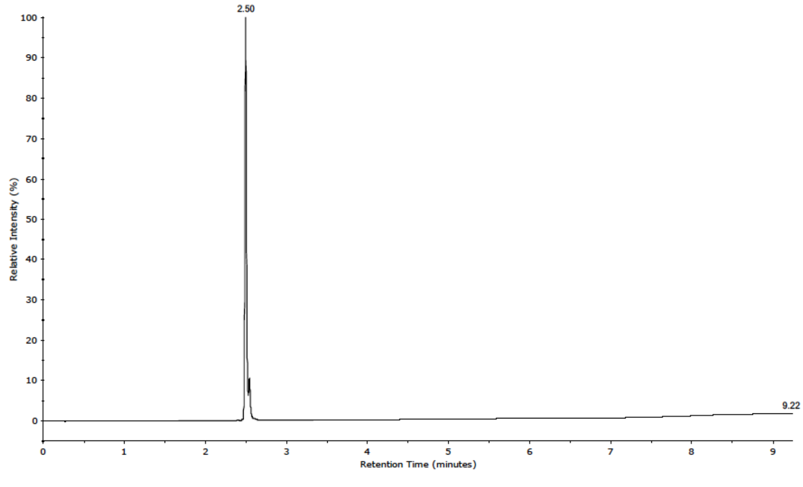

cyclo-CF[(4-Me)F)LYT

The peptide was synthesised using standard solid phase peptide synthesis techniques, yielding 5.9 mg (3.0%) as a white solid. LRMS (ESI$^+$): *m/z* 789.5, [M+H]$^+$, 1578.7 2[M+H]$^+$, 100%; HRMS (ESI$^+$) for $C_{41}H_{52}N_6O_8S$ found *m/z* 789.3636[M+H]$^+$; calculated 789.3640 (0.5 ppm error) and *m/z* 811.3452 [M+Na]+; calculated 811.3460 (-1.4 ppm error). HPLC retention time: 2.70 min.

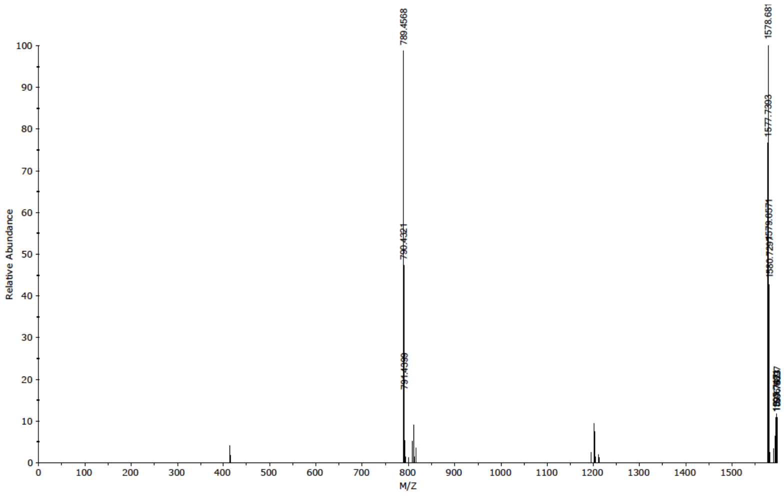

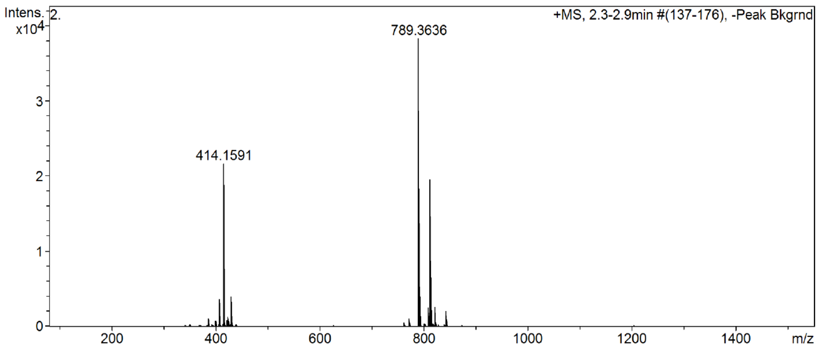

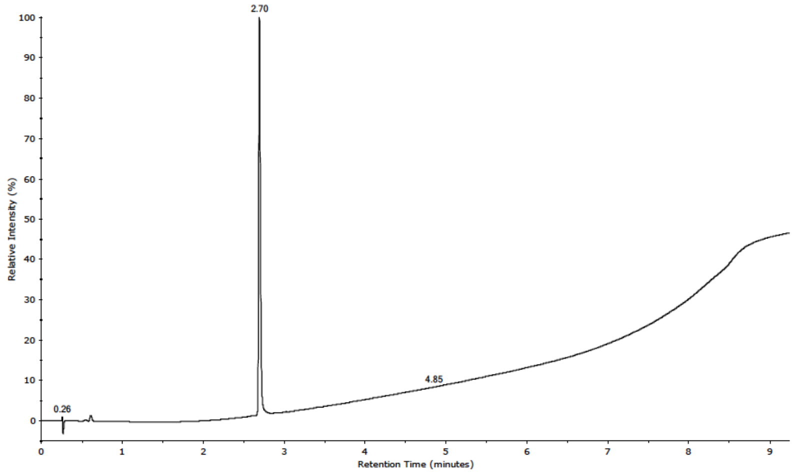

<u>*cyclo*-CF[(4-CN)F]LYT</u>

The peptide was synthesised using standard solid phase peptide synthesis techniques, yielding 10.7 mg (5.3%) as a white solid. LRMS (ESI$^+$): *m/z* 800.4, 100% [M+H]$^+$, 800.4, 78.78% [M+H]$^+$; HRMS (ESI$^+$) for $C_{41}H_{49}N_7O_8S$ found *m/z* 800.3431[M+H]$^+$; calculated 800.3436 (0.6 ppm error) and *m/z* 822.3245 [M+Na]$^{+;}$ calculated 822.3256 (1.2 ppm error). HPLC retention time: 2.35 min.

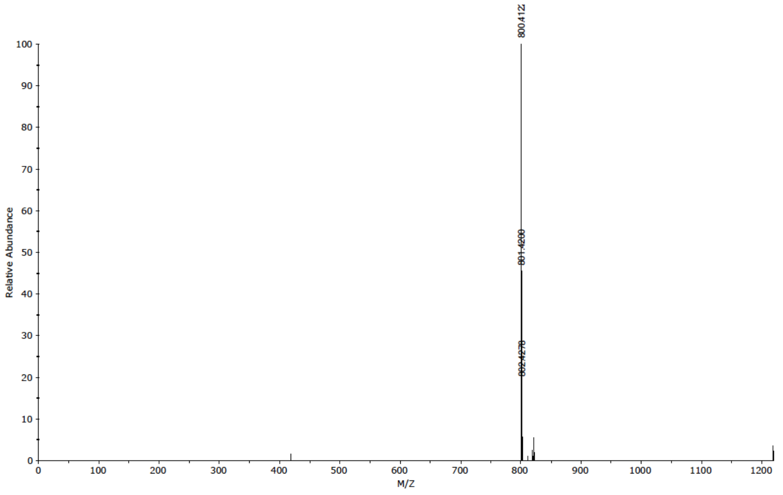

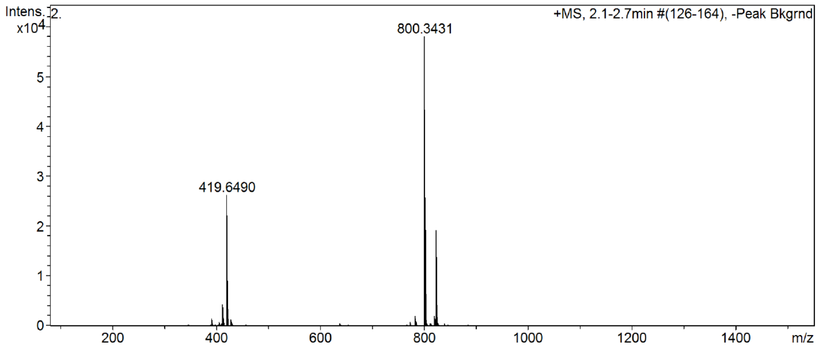

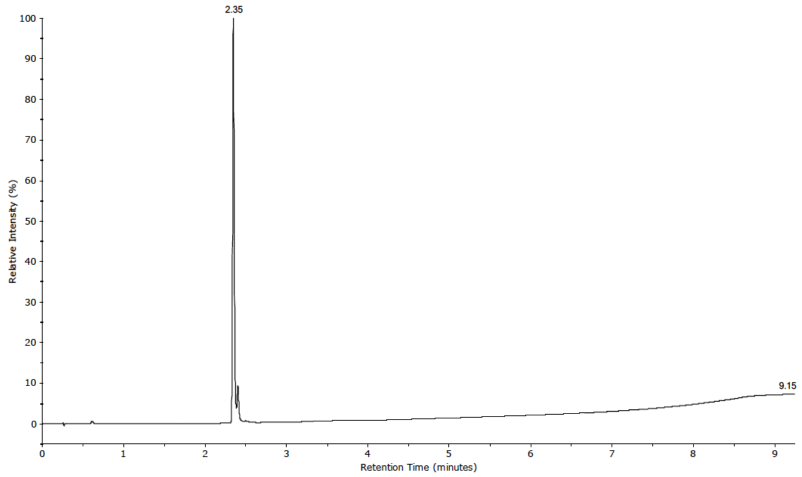

cyclo-CF[hF]LYT

The peptide was synthesised using standard solid phase peptide synthesis techniques, yielding 21.5 mg (10.9%) as a white solid. LRMS (ESI$^+$): *m/z* 789.4, 100% $[M+H]^+$; HRMS (ESI$^+$) for $C_{41}H_{52}N_6O_8S$ found *m/z* 789.3633 $[M+H]^+$; calculated 789.3640 (0.8 ppm error) and *m/z* 811.3460 (0.8 ppm error). HPLC retention time: 1.81 min.

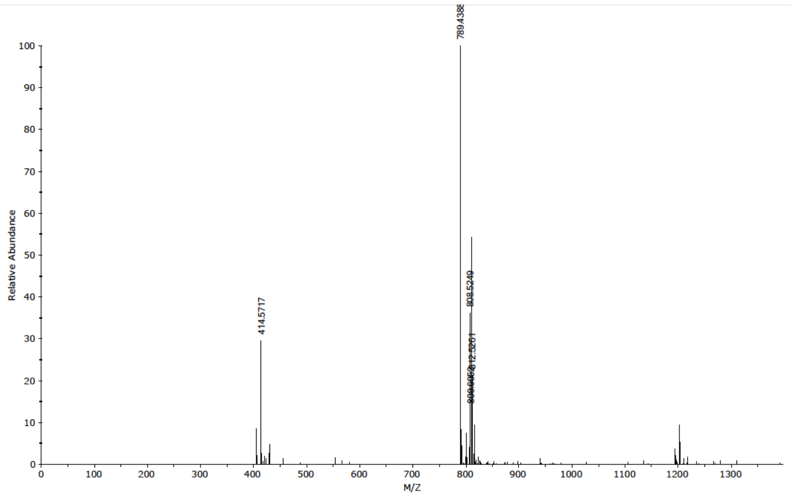

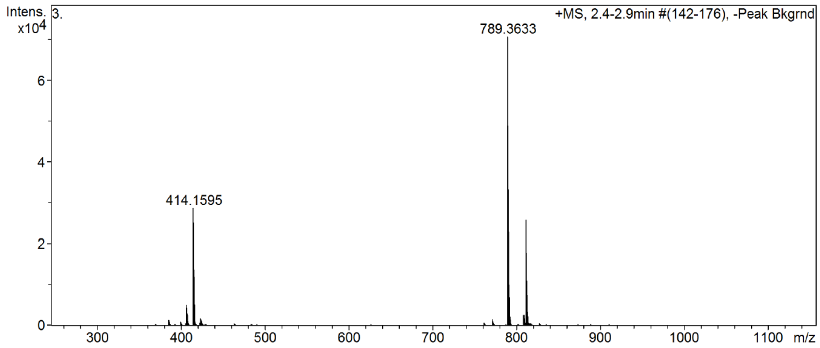

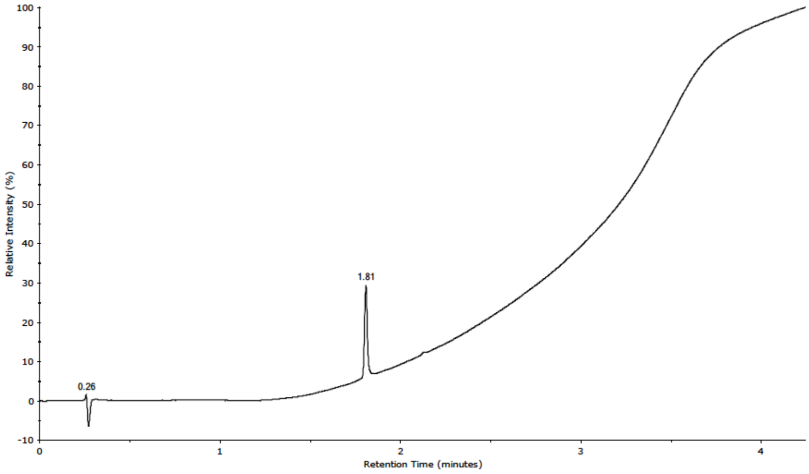

<u>*cyclo*-CF[1-Nal]LYT</u>

The peptide was synthesised using standard solid phase peptide synthesis techniques, yielding 18.3 mg (8.9%) as a white solid. LRMS (ESI$^+$): *m/z* 825.5, 94.33% [M+H]$^+$, 825.5, 100% [M+H]$^+$; HRMS (ESI$^+$) for $C_{44}H_{52}N_6O_8S$ found *m/z* 825.3633 [M+H]$^+$; calculated 825.3640 (0.8 ppm error) and *m/z* 847.3451 [M+Na]$^+$; calculated 847.3460 (1.0 ppm error). HPLC rentention time: 2.90 min.

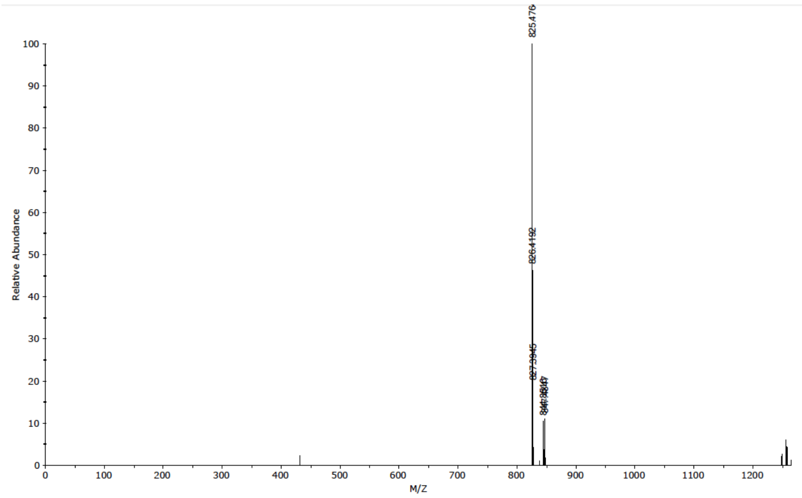

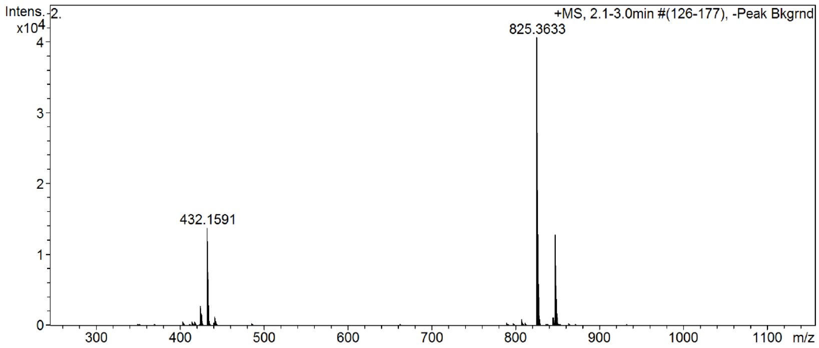

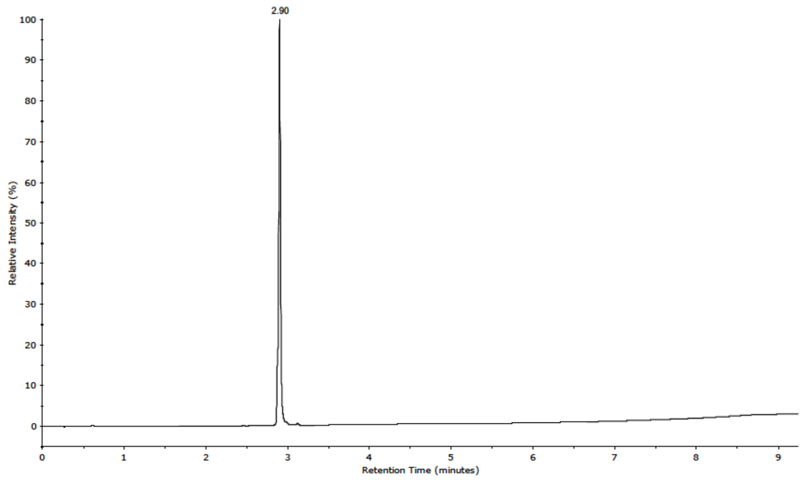

cyclo-CF[Cha]LYT (RINGPep2)

The peptide was synthesised using standard solid phase peptide synthesis techniques, yielding 25.0 mg (12.8 %) as a white solid. LRMS (ESI$^+$): *m/z* 781.8, 100% $[M+H]^+$. HRMS (ESI$^+$) for $C_{40}H_{56}N_6O_8S$ found *m/z* 781.3959 $[M+H]^+$; calculated 781.3953 (-0.8 ppm error). HPLC retention time: 2.12 min (RP-HPLC, 5 min, CH_3CN 20% to 100% in water).

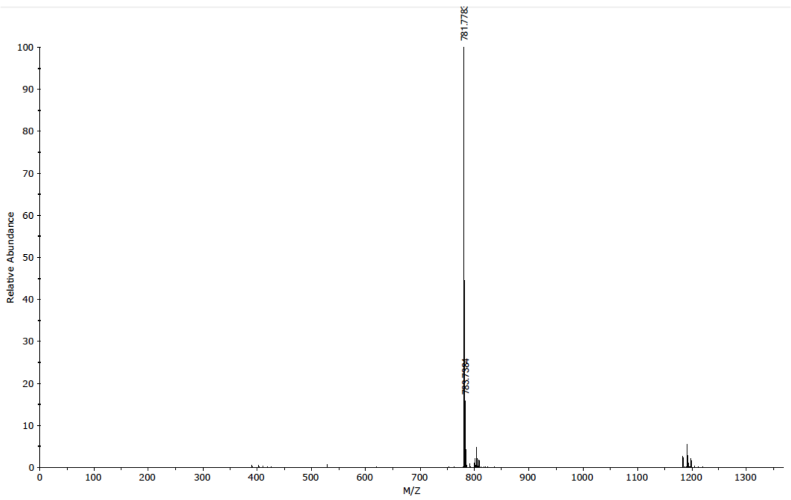

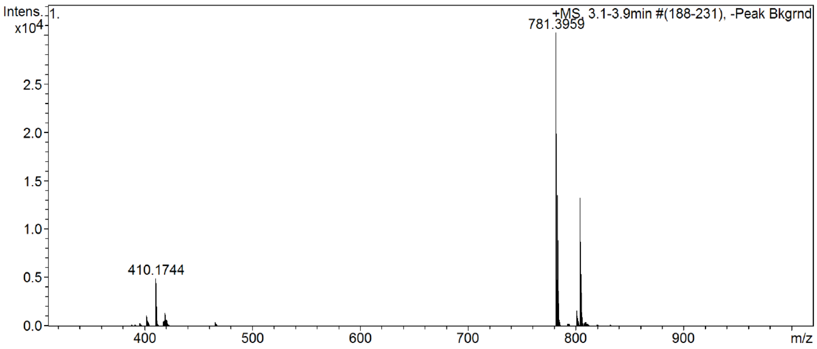

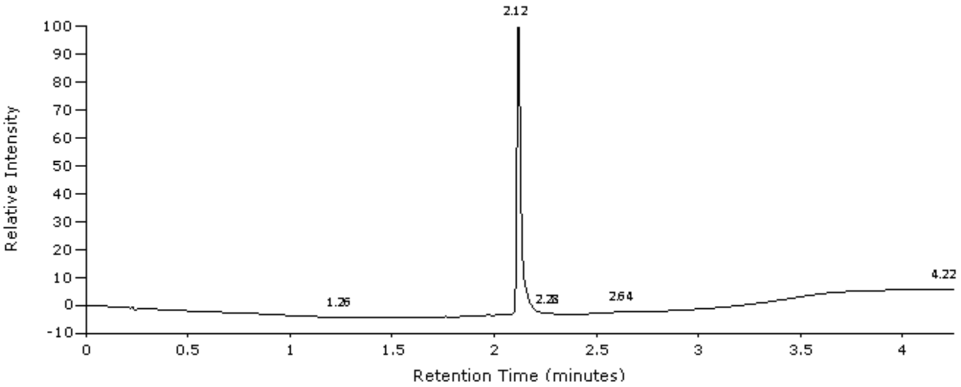

<u>*cyclo*-CYLFT-[Cha] (ScramPep2)</u>

The peptide was synthesised using standard solid phase peptide synthesis techniques, yielding 22.3 mg (5.7%) as a white solid. LRMS (ESI$^+$): *m/z* 781.8, 100% $[M+H]^+$. HRMS (ESI$^+$) for $C_{40}H_{56}N_6O_8S$ found *m/z* 781.3963 $[M+H]^+$; calculated 781.3953 (-1.2 ppm error). HPLC retention time 1.82 min.

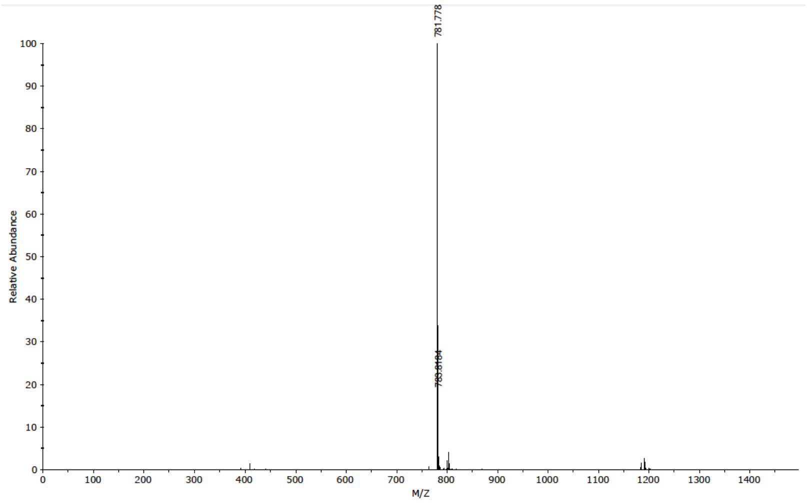

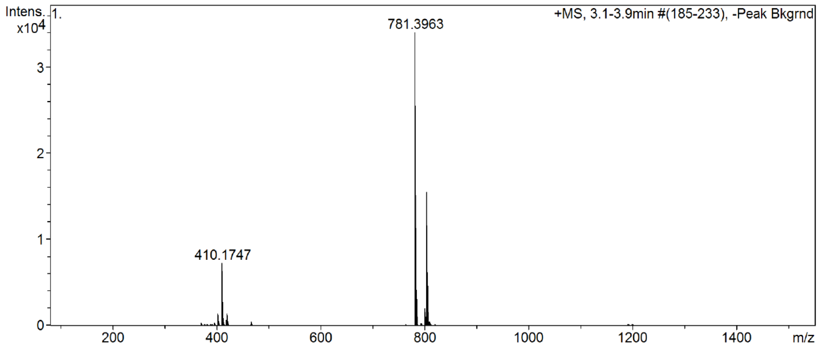

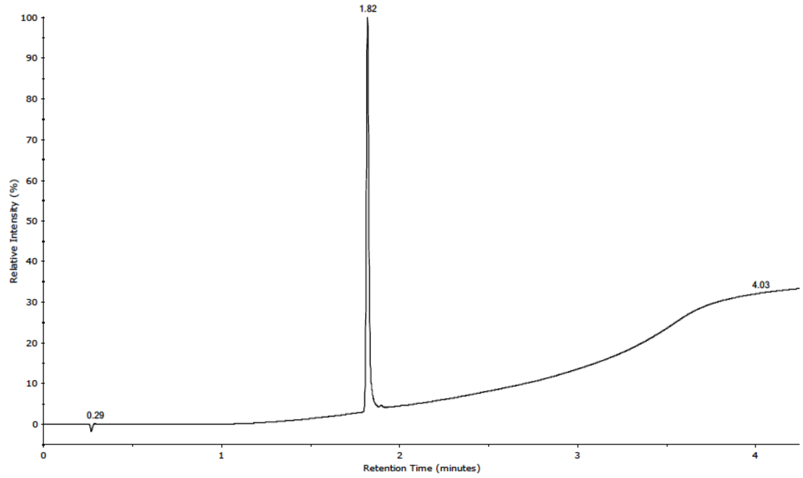

Tat-tagged *cyclo*-CF[Cha]LYT (Tat-RINGPep2)

The cyclic peptide was synthesised using standard solid phase peptide synthesis techniques. Following purification the Cys residue was protected with 2-aldrithiol (2 eq.) in DMF (1 mg/mL). The reaction was stirred for 24 h under argon at RT, and concentrated *in vacuo*. The resulting solution was added dropwise to 50 mL cold diethyl ether, resulting in precipitation of the cyclic peptide, which was purified by HPLC as above. The aldrithiol-protected cyclic peptides (1 eq.) were dissolved in DMF (1 mg/mL) and CGRKKRRQRRRPPQ (Tat-tag, 2 eq.) was added to the reaction dropwise. The reaction was stirred in an inert atmosphere for 24 h, followed by concentration *in vacuo*. The crude product was purified by HPLC as above and the product was characterized by mass spectrometry, yielding 6.6 mg (21%) as a white solid. Analytical HPLC (Acetonitrile/0.01% TFA and H_2O/0.01% TFA, 220 nm) 20.4 min (100%); LRMS (ESI$^+$): *m/z* 1302.2 $[M+2H]^+$, 868.2 $[M+3H]^+$, 651.5 $[M+4H]^+$, 521.5 $[M+5H]^+$, 434.7 $[M+6H]^+$, 372.5 $[M+7H]^+$, 100%. HRMS (ESI$^+$) not obtained as mass is above the detection threshold.

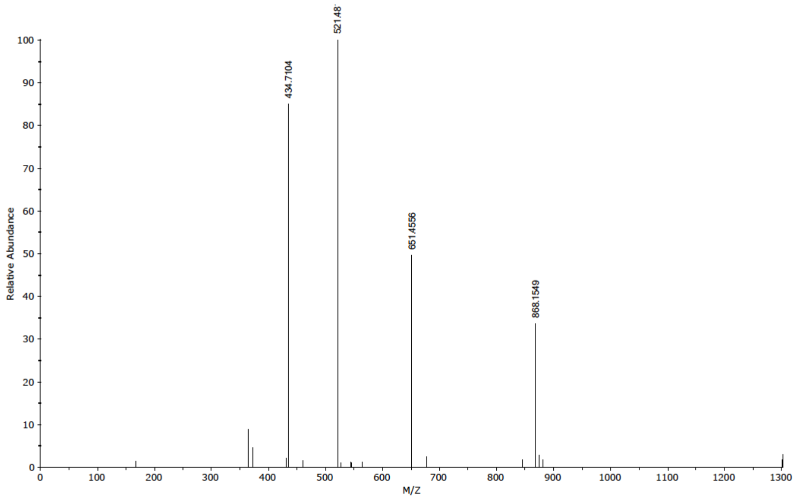

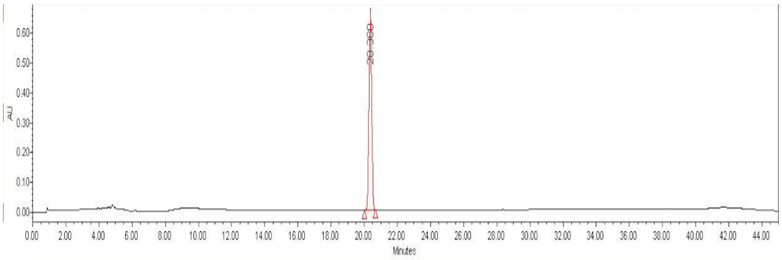

Supplement: Supplementary file 1 [file SC-009-C8SC01186A-s001.pdf]
